# *Phytophthora* methylomes are modulated by 6mA methyltransferases and associated with adaptive genome regions

**DOI:** 10.1186/s13059-018-1564-4

**Published:** 2018-10-31

**Authors:** Han Chen, Haidong Shu, Liyuan Wang, Fan Zhang, Xi Li, Sylvans Ochieng Ochola, Fei Mao, Hongyu Ma, Wenwu Ye, Tingting Gu, Lubin Jiang, Yufeng Wu, Yuanchao Wang, Sophien Kamoun, Suomeng Dong

**Affiliations:** 1 College of Plant Protection, Nanjing Agricultural University, Nanjing, 210095 China; 2 National Key Laboratory for Crop Genetics and Germplasm Enhancement, Nanjing Agricultural University, Nanjing, 210095 China; 3 College of Horticulture, Nanjing Agricultural University, Nanjing, 210095 China; 4 Institute Pasteur of Shanghai, Chinese Academy of Sciences, Shanghai, 200031 China; 5 The Sainsbury Laboratory, Norwich Research Park, Norwich, NR4 7UH UK

**Keywords:** *Phytophthora*, DNA methylation, Methyltransferases, Adaptive genome

## Abstract

**Background:**

Filamentous plant pathogen genomes often display a bipartite architecture with gene-sparse, repeat-rich compartments serving as a cradle for adaptive evolution. The extent to which this two-speed genome architecture is associated with genome-wide DNA modifications is unknown.

**Results:**

We show that the oomycetes *Phytophthora infestans* and *Phytophthora sojae* possess functional adenine N6-methylation (6mA) methyltransferases that modulate patterns of 6mA marks across the genome. In contrast, 5-methylcytosine could not be detected in these species. Methylated DNA IP sequencing (MeDIP-seq) of each species reveals 6mA is depleted around the transcription start sites (TSSs) and is associated with lowly expressed genes, particularly transposable elements. Genes occupying the gene-sparse regions have higher levels of 6mA in both genomes, possibly implicating the methylome in adaptive evolution. All six putative adenine methyltransferases from *P*. *infestans* and *P*. *sojae*, except PsDAMT2, display robust enzymatic activities. Surprisingly, single knockouts in *P*. *sojae* significantly reduce in vivo 6mA levels, indicating that the three enzymes are not fully redundant. MeDIP-seq of the *psdamt3* mutant reveals uneven 6mA methylation reduction across genes, suggesting that PsDAMT3 may have a preference for gene body methylation after the TSS. Furthermore, transposable elements such as DNA elements are more active in the *psdamt3* mutant. A large number of genes, particularly those from the adaptive genomic compartment, are differentially expressed.

**Conclusions:**

Our findings provide evidence that 6mA modification is potentially an epigenetic mark in *Phytophthora* genomes, and complex patterns of 6mA methylation may be associated with adaptive evolution in these important plant pathogens.

**Electronic supplementary material:**

The online version of this article (10.1186/s13059-018-1564-4) contains supplementary material, which is available to authorized users.

## Background

DNA methylation, one of the fundamental epigenetic marks, participates in many biological processes in both eukaryotes and prokaryotes [1–3]. The most studied form of DNA methylation is 5-methylcytosine (5mC), which is a prevalent DNA modification in mammals and plants [4]. The 5mC modification plays a role in many processes, such as transposon silencing, regulation of gene expression, and epigenetic memory maintenance [5]. The amount of 5mC present in DNA varies across organisms and is barely detectable or absent in many species, such as the nematode (*Caenorhabditis elegans*), the fruit fly (*Drosophila melanogaster*), and brewer’s yeast (*Saccharomyces cerevisiae*) [6]. Comparatively, the N6-methyladenine (6mA) modification is extensively distributed in prokaryotic genomes. A prominent function of 6mA is in discriminating between host DNA and invading DNA, thus contributing to prokaryote immunity against phages and other invading genetic elements [7]. Besides, 6mA is also involved in DNA replication, repair, virulence, and gene regulation [8–11].

In contrast to prokaryotes, the occurrence and biological functions of 6mA methylation in eukaryotic organisms remain largely uncharacterized. There is increasing evidence that 6mA is present in eukaryotes, including mammals, nematodes, algae, fruit flies, frogs, and fungi [12–16]. Genome-wide 6mA distribution patterns can be identified by several robust methods such as methylated DNA immunoprecipitation sequencing (MeDIP-seq) [14, 15], 6mA-sensitive restriction enzyme digestion coupled with high-throughput sequencing [17], and single-molecule real-time sequencing (SMRT sequencing) [12, 13]. The 6mA pattern appears to be dynamic during development; for instance, the early embryonic stage of *Drosophila* has relatively higher 6mA levels compared to later stages [14, 18]. Furthermore, the genomic localization of 6mA significantly differs among organisms [13–15]. The 6mA modification is widely and evenly distributed in the *Caenorhabditis elegans* genome. By contrast, 6mA is enriched around transcription start sites (TSSs) in early-diverging fungi and *Chlamydomonas* and is enriched in transposable elements in *Drosophila*. The localization patterns associate with 6mA biological functions. For example, in *Chlamydomonas* and fungi, 6mA is enriched around the TSSs of actively expressed genes, suggesting that 6mA may be an active mark for gene expression [12, 15], while 6mA appears to suppress transcription on the X chromosome in mouse embryonic stem cell [16].

Previous studies have demonstrated that 6mA could be reversibly modulated by enzymes such as methyltransferase and demethylase [13, 14]. It is known that DAM and M.MunI are classical bacterial 6mA methyltransferases [19]. In eukaryotic cells, enzymes from the MT-A70 protein family that evolved from M.MunI [20] are considered 6mA methyltransferases. Overexpression of the MT-A70 homolog DAMT-1 from *C*. *elegans* in insect cells elevated the 6mA level, whereas knockdown of *damt-1* resulted in a decrease in the amount of 6mA, suggesting that DAMT-1 is a potential 6mA methyltransferase in nematodes [13]. However, methyltransferase-like protein 3 (METTL3) and METTL14 of the MT-A70 family catalyze 6mA on mammalian mRNA but weakly on DNA [21]. The alkylation repair homologs (AlkB) protein family is involved in DNA damage repair and could catalyze demethylation of both methylated DNA and RNA [13, 16, 22, 23]. MT-A70 and AlkB homologs are prevalent in many organisms, and most of them are not functionally characterized. However, it is possible that other RNA and DNA demethylase and methyltransferase proteins could have evolved to regulate 6mA DNA in eukaryotic species.

The Oomycetes are a group of eukaryotic organisms that include numerous pathogens that infect plants and animals [24]. A notorious example is *Phytophthora infestans*, the causal agent of potato late blight disease which sparked the Irish famine, resulting in starvation and migration of millions of people in the 1840s [25]. An additional example is *Phytophthora sojae*, a soybean root pathogen that currently threatens global soybean production. These two species are model organisms among oomycetes [26]. The genomes of these *Phytophthora* display a bipartite architecture, with gene-sparse and repeat-rich regions (GSR) and gene-dense regions (GDR) [25]. The GSR compartments are associated with accelerated gene evolution, serving as a cradle for adaptive evolution [27–29]. However, the biological roles of DNA modifications and their associations with adaptive genome evolution remain unknown. This study demonstrates that 6mA, rather than 5mC, is the major DNA methylation in these two *Phytophthora* species. We show that *P*. *infestans* and *P*. *sojae* genomes encode expanded numbers of 6mA methyltransferases (DAMT) with varied catalytic activity. The 6mA methylation landscapes are described at the genome-wide level using methylated DNA immunoprecipitation sequencing (MeDIP-seq). Although the majority of the methylation sites localize in the intergenic regions, 6mA also prefers to accumulate around TSS regions in a bimodal distribution pattern and tends to associate with lowly expressed or silenced genes. The GSR genes show higher methylation level than the GDR genes. Consistently, most 6mA sites accumulate in repetitive sequences, such as DNA elements and long terminal repeat (LTR) elements. Furthermore, individual knockouts of each of the three *DAMT* genes result in a reduction of 6mA level in vivo. *psdamt3* mutant showed normal mycelium growth but significantly impaired virulence on susceptible plants. Further MeDIP-seq analysis confirmed a global 6mA reduction, including reduction in both transposable elements and methylated gene locus. A large proportion of genes in particular genes from adaptive genome compartment are differentially expressed in *psdamt3* mutant. These results suggest that the DAMTs may have functional specificity in targeting particular genomic regions and participate in the interactions with host plants.

## Results

To determine whether *Phytophthora* species can accomplish the 5mC modification, we performed a hidden Markov model-based sequence similarity search for 5mC methyltransferase homologs in the *P*. *infestans* and *P*. *sojae* genomes [30, 31]. No predicted gene or homologous sequence corresponding to a 5mC methyltransferase was discovered (Additional file 1: Table S1). To test directly for the presence of 5mC, we analyzed hydrolyzed genomic DNA (gDNA) samples from *P*. *infestans* and *P*. *sojae* by high-performance liquid chromatography (HPLC) and ultra performance liquid chromatography electrospray ionization-mass spectrum (UPLC-ESI-MS/MS). We did not detect 5mC in either species at the parts per billion (PPB) level (10^−9^ g/mL) (Additional file 2: Figure S1a, b). Furthermore, the endonuclease McrBC that specifically cleaves DNA containing 5mC did not digest *Phytophthora* gDNA, similar to gDNA of *Drosophila melanogaster*, which is known not to carry 5mC (Additional file 2: Figure S1c). Therefore, none of the methods we employed could detect 5mC in either *P*. *infestans* or *P*. *sojae* DNA.

**Fig. 1:**
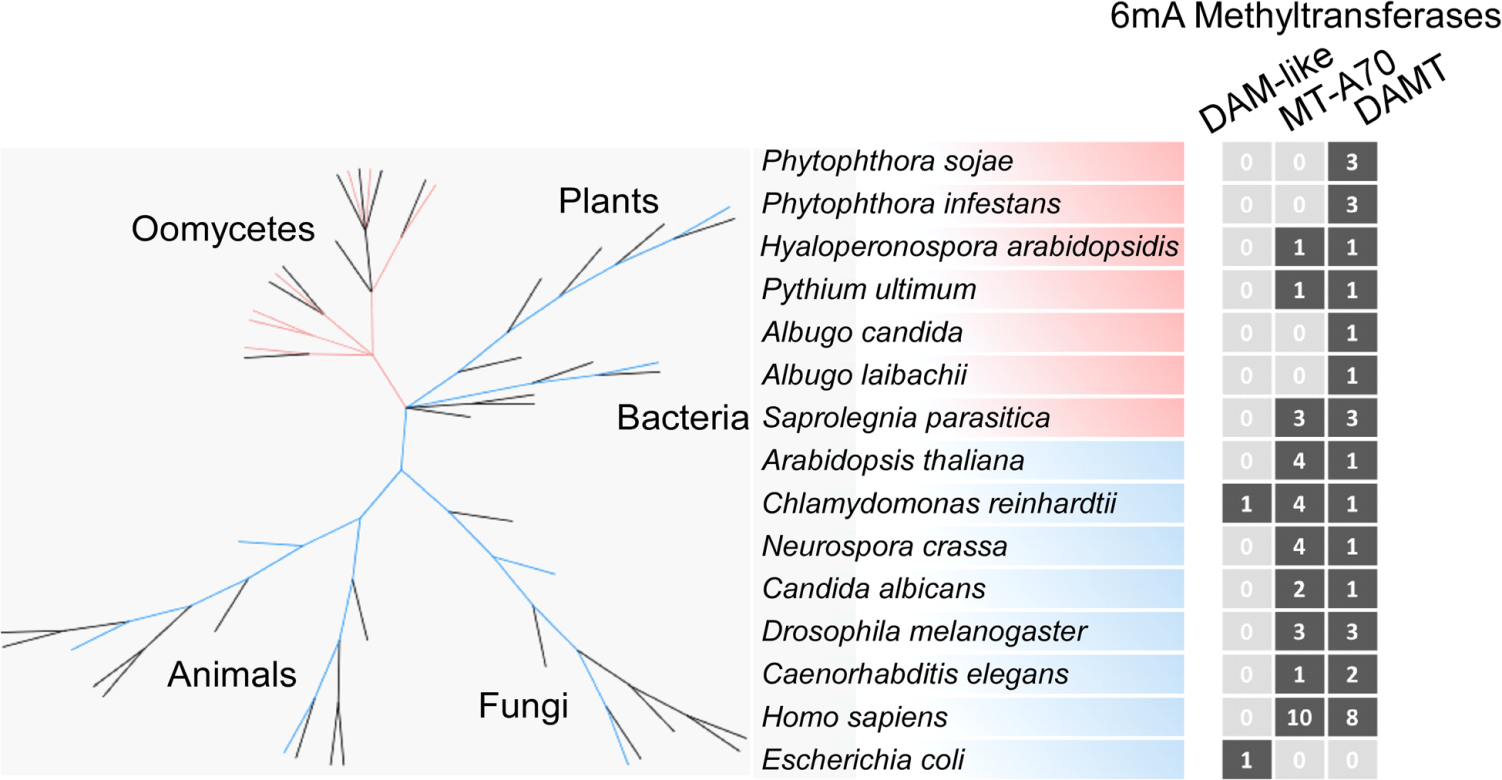
*Phytophthora* genomes encode 6mA methyltransferases. N6-adenineMlase domain-containing (*DAMT*) gene expansion in *Phytophthora* species. Two families of methyltransferase genes (*DAM-like*, *MT-A70*) and the *DAMT* family genes from seven oomycete organisms and eight model organisms are shown in a simplified phylogenetic tree. Color codes: oomycetes (red), other model organisms (blue), presence of gene homolog (black), and absence of gene homolog (gray). The number of homologous genes in each organism is also labeled

Although we did not identify genes encoding for 5mC methyltransferases in *P. infestans* and *P. sojae*, we did identify homologs of 6mA methyltransferases and demethylases in the *Phytophthora* genomes. Initially, we discovered a potential MT-A70 homolog in the *P. sojae* but not *P. infestans* genome. However, a closer examination of the putative *P. sojae* MT-A70 gene indicated that it is a pseudogene with a premature stop codon. We found that N6-adenineMlase domain-containing (*DAMT*) proteins are present in all the examined oomycete species, including *Phytophthora* species, *Albugo* species, *Hyaloperonospora arabidopsidis*, *Pythium ultimum*, and *Saprolegnia parasitica* (Fig. 1). The *Phytophthora* and *Saprolegnia* genomes each encode three predicted *DAMT* genes, whereas the other species have only one gene. Phylogenetic analyses of the oomycete DAMTs uncovered two distinct gene clades, namely DAMT1/2 and DAMT3 (Additional file 2: Figure S2a). In contrast to *DAMT1* and *DAMT2*, *DAMT3* is conserved in all the examined oomycete genomes except *H. arabidopsidis* (Additional file 2: Figure S2a, Additional file 3: Table S2). *DAMT3* is located in a genomic region with a high degree of synteny (Additional file 2: Figure S2b), suggesting that it is probably the ancestral gene. *DAMT* gene expansion in *Phytophthora* species, therefore, appears to be due to the emergence of the *DAMT1/2* genes. A closer examination of the catalytic motif responsible for binding the methyl group from *S*-adenosyl-l-methionine [13, 20, 32] indicates that DAMT1 and DAMT3 proteins have functional motifs consisting of the amino acid sequences DPPY and DPPF, respectively. However, this motif was naturally mutated into EPPH in the DAMT2 proteins. A search in the *P. infestans* and *P. sojae* online RNA-seq databases revealed that *DAMTs* are expressed in all the examined growth stages [33, 34] (Additional file 2: Figure S2d, e). In summary, bioinformatics analyses indicate that *Phytophthora* species may possess the enzymatic machinery for 6mA DNA methylation.

To verify the enzymatic activity of these putative methyltransferases, we measured the in vitro methyltransferase activity of recombinant DAMT proteins. The recombinant proteins, together with 6mA-free lambda DNA and substrate *S*-adenosyl-l-methionine, were incubated together in an in vitro enzymatic assay [35]. These assays revealed that lambda DNA is smeared by treatment with the restriction enzyme DpnI, which recognizes the 6mA methylated GATC site, in the presence of recombinant PsDAMT1, PsDAMT3, PiDAMT1, PiDAMT2, PiDAMT3, or the bacterial 6mA methyltransferase DAM (Additional file 2: Figure S3a). We did not detect any activity of PsDAMT2 in this assay, even after increasing PsDAMT2 concentration (Additional file 2: Figure S3a). We also performed a complementary methylation assay in the 6mA-deficient *Escherichia coli* strain HST04. In this assay, *E. coli* gDNA from DH5α and *DAM*-complemented HST04 transformants were digested by DpnI as expected. The *E. coli* gDNA from *PsDAMT1* and *PsDAMT3* transformants could also be digested by DpnI, whereas those from *PsDAMT2* and the transformants of the catalytically dead mutants (*PsDAMT1*^*APPA*^, *PsDAMT3*^*APPA*^) could not be digested (Additional file 2: Figure S3b). Overall, these data indicate that the DAMT proteins possess methyltransferase activity in two *Phytophthora* species.

**Fig. 2:**
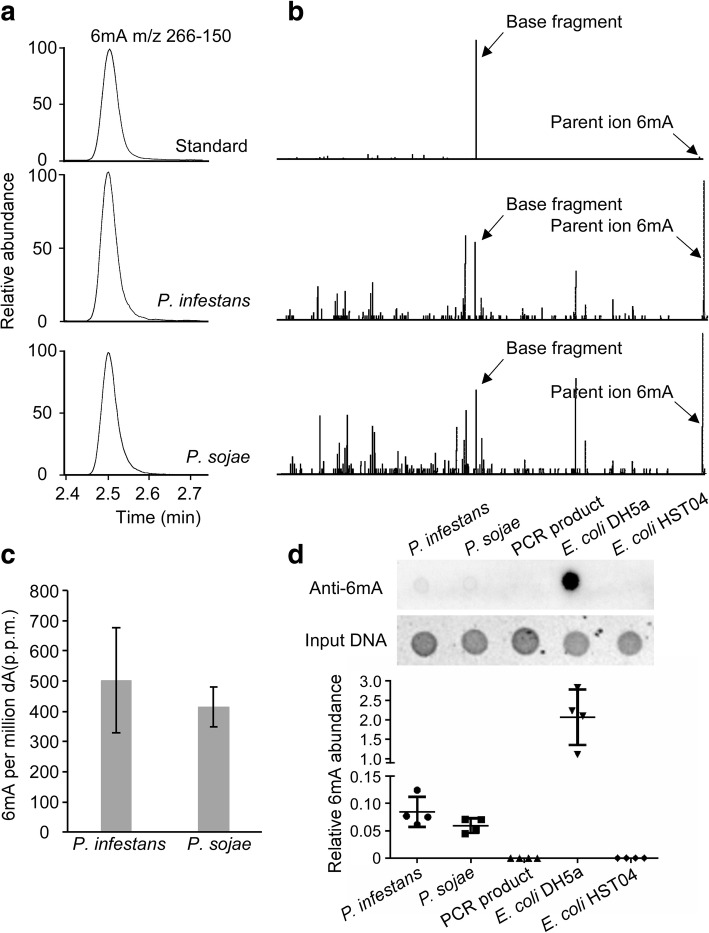
6mA occurs in *Phytophthora* genomic DNA. **a** *Phytophthora* mycelium 6mA were detected by UPLC. The selective multiple reaction monitoring (MRM) transitions for 6mA were setting as *m*/*z* 266–150. The retention time of standard 6mA was present in *Phytophthora* gDNA samples. **b** *Phytophthora* 6mA are detected by UPLC-ESI-MS/MS. The parent ion 6mA (*m*/*z* near 266.12) and base fragment (*m*/*z* near 150.07) highlighted by arrows from samples also match standard 6mA. **c** Quantification of 6mA levels in *Phytophthora* samples. The 6mA concentrations are listed as 6mA per million dA. **d** The presence of 6mA in *Phytophthora* gDNA was verified by dot blot assay using a specific 6mA antibody. Input DNA was quantified by ethidium bromide-dyed agarose gels. Every dot loaded 100 ng DNA. The experiments were independently carried out in four replicates. The relative 6mA abundance is calculated (integrated signal density_anti-6mA_/integrated signal density_input DNA_) with the signal density quantified by ImageJ

To test for the presence of 6mA in *Phytophthora*, we used UPLC-ESI-MS/MS to analyze gDNA samples from *P. sojae* and *P. infestans*. A peak matching the retention time of standard 6mA was present in the test samples from these two *Phytophthora* species (Fig. 2a). Moreover, the same base fragment was detected in the two samples by MS/MS of 266.12 (mass/charge ratio), which also matched the standard 6mA (Fig. 2b). Thus, the 6mA DNA base modification is present in the gDNA samples. Moreover, we estimated the abundance of 6mA in *P. sojae* and *P. infestans* to be 400 and 500 parts per million (PPM), respectively, as determined by UPLC-ESI-MS/MS (Fig. 2c, Additional file 4: Table S3). The 6mA level in these *Phytophthora* species was approximately 60-fold higher than that in *Homo sapiens* and *Mus musculus* but was lower than that in a few early-diverging fungal species like *Hesseltinella vesiculosa* and *Piromyces finnis* [12, 36]. To further test for the presence of 6mA in *Phytophthora* gDNA, we used commercially available antibodies that specifically recognize the 6mA modification; immune blot signals were robustly detected in gDNA samples of *P. infestans* and *P. sojae* (Fig. 2d). Collectively, our results show that 6mA is a naturally occurring DNA modification in *Phytophthora* genomes.

We performed methylated DNA immunoprecipitation sequencing (MeDIP-seq) to obtain a genome-wide insight into the *Phytophthora* 6mA methylome [37]. The MeDIP-seq experiments on gDNA samples from mycelium growth stages included two biological replicates for each of the two *Phytophthora* species. After assembling sequencing data and seeking 6mA-enriched regions, we mapped 6mA peaks (6mA-enriched regions) at a genome-wide level with FDR < 0.01 by SICER [38]. A total of 12,611 overlapping methylation peaks were captured from the two *P. infestans* biological replicates. A total of 3031 overlapping peaks were called from two *P. sojae* replicates (Additional file 2: Figure S4a). Genome-wide 6mA methylation profiling data revealed that 86% and 55% of the 6mA peaks were located in the intergenic regions in *P. infestans* and *P. sojae*, respectively (Additional file 2: Figure S4b). The higher proportion of 6mA intergenic localization in *P. infestans* resulted from the larger overall fraction of intergenic gDNA in the expanded 240 Mbp genome of this species compared to *P. sojae*. In *P. sojae*, 25% of the 6mA peaks mark gene bodies, whereas 15% and 5% of the methylations occupy positions upstream and downstream of gene bodies, respectively. Comparatively, in *P. infestans*, these figures correspond to 8%, 4%, and 2% (Additional file 2: Figure S4b). Overall, our analysis revealed 1805 and 1343 genes with 6mA marks in *P. infestans* and *P. sojae*, respectively.

**Fig. 3:**
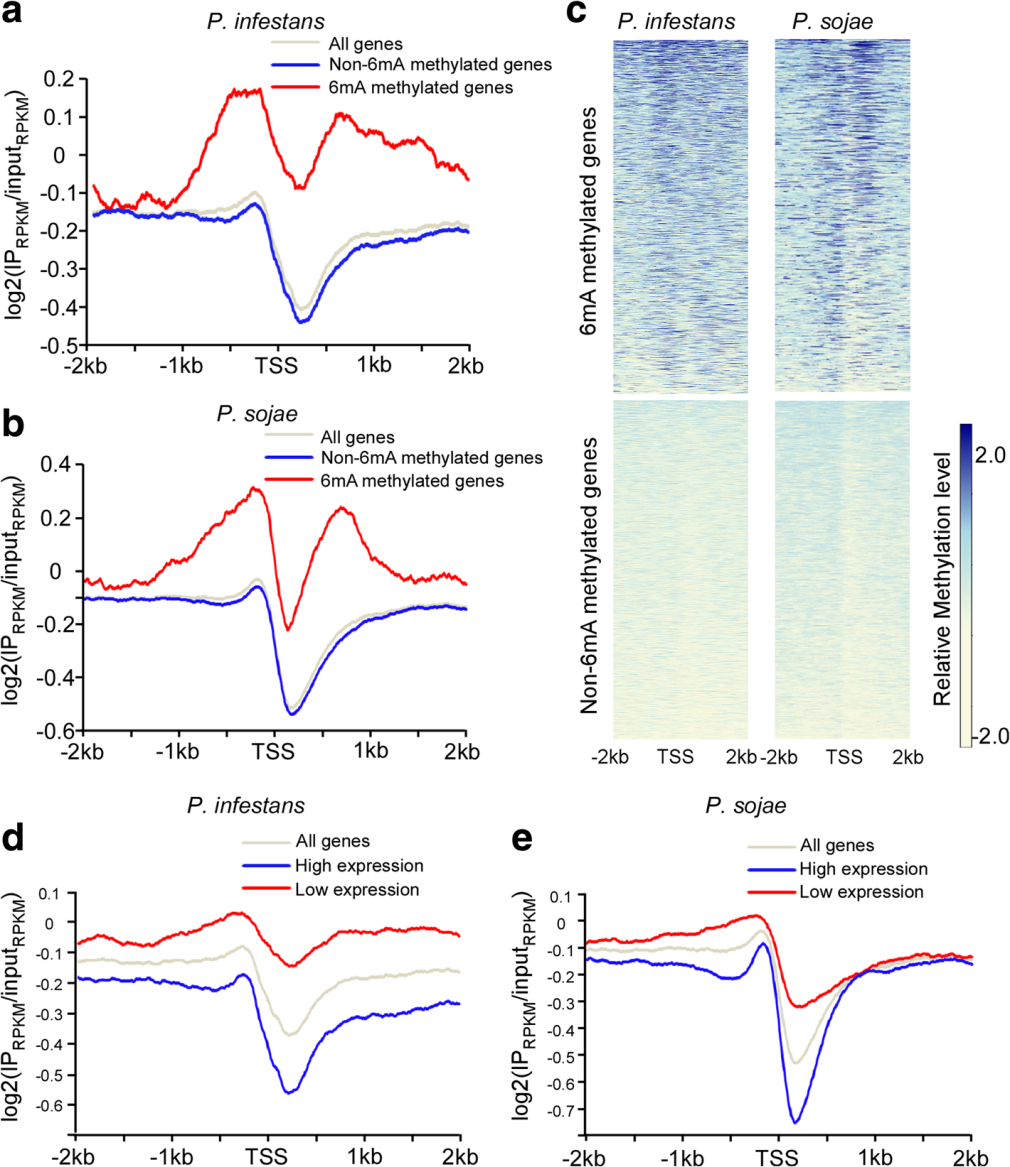
Bimodal distribution pattern of 6mA around TSS in *Phytophthora*. The distribution of 6mA peaks around TSS was profiled by MeDIP-seq. The 6mA occupancy along TSS from − 2 to 2 kb is shown. 6mA peaks were enriched around TSS with a bimodal distribution and a local depletion after TSS in *P*. *infestans* and *P*. *sojae*. **a** 6mA occupancy in *P*. *infestans* methylated genes. All *P. infestans* genes are divided into 6mA methylated genes (*n* = 1805) and non-6mA methylated genes (*n* = 16374). **b** 6mA occupancy in *P*. *sojae* methylated genes. All *P*. *sojae* genes are divided into 6mA methylated genes (*n* = 1343) and non-6mA methylated genes (*n* = 17853). **c** Heatmap analyses of 6mA signal from individual genes verified the bimodal distribution pattern in both *Phytophthora* species. The relative methylation signal is represented using gradient colors. **d** The 6mA level is negatively correlated with gene expression in *P*. *infestans*. All genes are divided into two groups: high expression (FPKM ≧ 5, *n* = 9927) and low expression (FPKM < 5, *n* = 8252). **e** The 6mA level is negatively correlated with gene expression in *P*. *sojae*. All genes are divided into two groups: high expression (FPKM ≧ 5, *n* = 9450) and low expression (FPKM < 5, *n* = 9746)

Profiling of 6mA distribution in methylated genes revealed that 6mA peaks tend to flank the transcriptional start site (TSS) with a clear depletion near the TSS itself (Fig. 3a–c), resembling the bimodal distribution pattern of 6mA detected in other organisms, such as *Chlamydomonas* [15]. To investigate whether AT content around TSS might cause bias, we calculated the percentage of AT content around the TSS region (Additional file 2: Figure S5a). A slight AT content change around TSS was observed; we reason that this could be caused by start codon usage. Although weak AT change is present, it does not correlate with the dual methylation peaks around TSS. This data indicated that the enriched methylation signal is reliable. This bimodal distribution pattern could be verified by heatmap analyses when we plot relative 6mA levels from all the methylated and non-methylated genes (Fig. 3c). We illustrate normalized 6mA MeDIP-seq reads mapped onto loci from *P. sojae Ps_155563* and *Ps_128235* and *P. infestans PITG_02506*, *PITG_02507*, and *PITG_15808* as typical examples of 6mA localization patterns (Additional file 2: Figure S5b). To gain further insight into the characteristics of 6mA methylated genes, we conducted a gene ontology (GO) enrichment analysis of methylated genes in both species (Additional file 5: Tables S4–S9). Results from the GO analysis suggest that methylated genes are associated with functional categories, such as chromatin binding, enzyme regulator, and enzyme activity (Additional file 5: Tables S4, S6, and S9).

Although it is debatable how well a GO analysis can inform questions of biological function, there is increasing evidence that 6mA is an important epigenetic mark for the regulation of gene expression [12, 15]. In particular, the bimodal localization of the 6mA signal around the TSS prompted us to investigate the relationship between 6mA modification and gene expression. We compared the 6mA gene methylation data with RNA-seq gene expression data [39] and examined the average 6mA level of highly expressed genes (FPKM ≧ 5) and lowly expressed genes (FPKM < 5) in *P. infestans* and *P. sojae*. Lowly expressed genes are more likely to be associated with 6mA as this group of genes tends to have more abundant 6mA levels; in contrast, highly expressed genes tend to have lower 6mA levels (Fig. 3d, e). To further validate these observations, we examined the gene expression levels of methylated and non-methylated genes in both species. We found that methylated genes have significantly lower gene expression compared to non-methylated genes in both species (Additional file 2: Figure S6a, b). Thus, the data suggest that 6mA negatively correlates with gene expression levels in the two *Phytophthora* species.

**Fig. 4:**
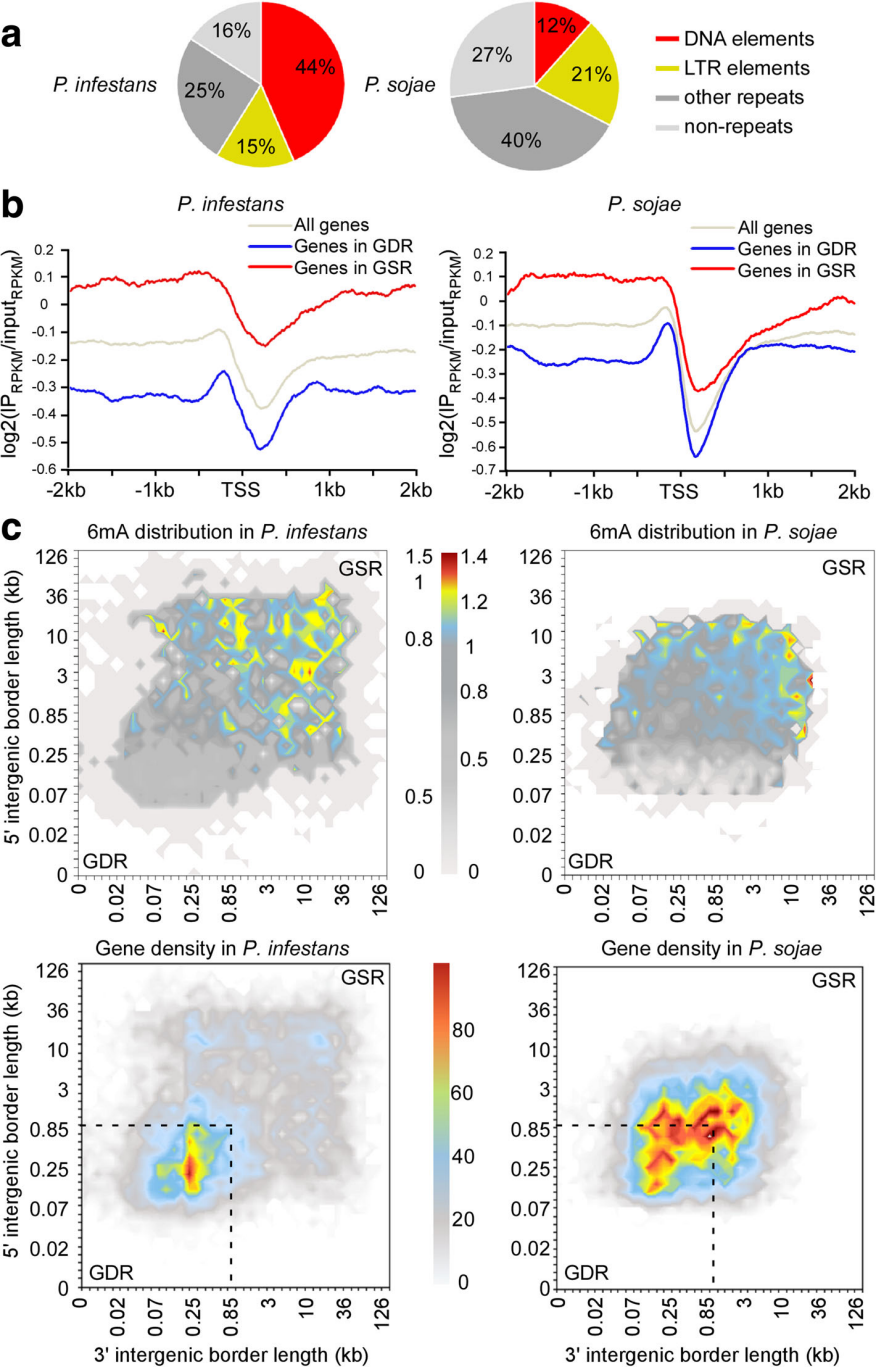
6mA marks *Phytophthora* repetitive elements and highlights gene-sparse region. **a** Pie charts illustrate that 6mA peaks are predominantly distributed in repeat sequences in *P*. *infestans* and *P*. *sojae.* **b** Genes occupying the gene-sparse regions (GSR) display higher levels of 6mA than genes occupying the gene-dense region (GDR) in *P*. *infestans* and *P*. *sojae*. In *P*. *infestans*, GSR-occupying genes (*n* = 3920) and GDR-occupying genes (*n* = 6526) were calculated. In *P*. *sojae*, GSR-occupying genes (*n* = 3154) and GDR-occupying genes (*n* = 7240) were calculated. **c** Heatmap analyses reveal that 6mA accumulate in *P*. *infestans* and *P*. *sojae* GSR. All the genes in each genome were sorted into two-dimensional bins on the basis of the lengths of flanking intergenic distances to neighboring genes at their 5′ and 3′ ends. For the 6mA distribution heatmap, gradient color represents the average normalized 6mA value of genes in each bin. For the gene density distribution heatmap, gradient color represents the number of genes in each bin. The dotted line highlights GDR

It is well established that genomes of *Phytophthora* species have experienced repeat-driven expansions and are, therefore, rich in repetitive sequences [25–28]. Thus, we examined the association between 6mA peaks and major types of transposable elements (TEs). A total of 37% (*P. infestans*) and 15% (*P. sojae*) of the 6mA peaks locate to long terminal repeat (LTR) elements (class I TEs), whereas 8% (*P. infestans*) and 10% (*P. sojae*) of the peaks fall within DNA elements (class II TEs), respectively (Fig. 4a). Statistical analyses indicate that 6mA peaks are enriched in TEs at a significant level (Additional file 2: Figure S7a)*.* Moreover, 6mA levels in TEs are higher than the average genomic level in both species at DNA elements (Additional file 2: Figure S7b)*.* We conclude that the 6mA methylome is preferentially associated with TEs in the two *Phytophthora* species.

The genomes of *Phytophthora* species have a bipartite “two-speed” architecture with distinct GDR and GSR [25–28]. The dynamic GSRs are enriched in rapidly evolving genes, such as virulence effectors, and these regions are thought to enable a faster rate of pathogen evolution [27–29]. To investigate the relationship between 6mA and genome architecture, we calculated the average 6mA levels for genes located in GDR and GSR. These analyses revealed that genes in the GSR tend to have a higher 6mA level than GDR genes (Fig. 4b, c). In *P. infestans*, AT contents between GDR and GSR were very similar, suggesting that the stronger 6mA signals from GSR genes were not due to AT content bias. In *P. sojae*, a slightly higher AT content of GSR gene locus was observed, but clearly, the enriched 6mA signals from − 200 bp to TSS were not due to AT bias (Additional file 2: Figure S8). Similarly, we plotted the 6mA methylation RPKM value of the region corresponding to the 500 bp after the TSS according to local gene density (measured as the length of 5′ and 3′ flanking intergenic regions) to generate the genome architecture heatmaps as previously described [25, 27]. The heatmaps revealed a clear association between the methylome and genome architecture that genes with higher 6mA levels were enriched in the GSR and reduced in GDR (Fig. 4c, Additional file 2: Figure S9a). These observations are consistent with our previous finding that 6mA preferentially accumulate in repetitive and TE-rich regions, which fill the intergenic regions in the GSR of *Phytophthora* genomes. Interestingly, further MeDIP-seq analyses demonstrated that secretome genes, including RxLR effector genes, which are important in *Phytophthora*-host interactions and were primarily localized in the GSR, had significantly higher 6mA levels than core orthologous genes (Additional file 2: Figure S9b, c). We conclude that the 6mA methylome was preferentially associated with both the genes and intergenic regions that form the gene-sparse compartments of *Phytophthora* genomes.

**Fig. 5:**
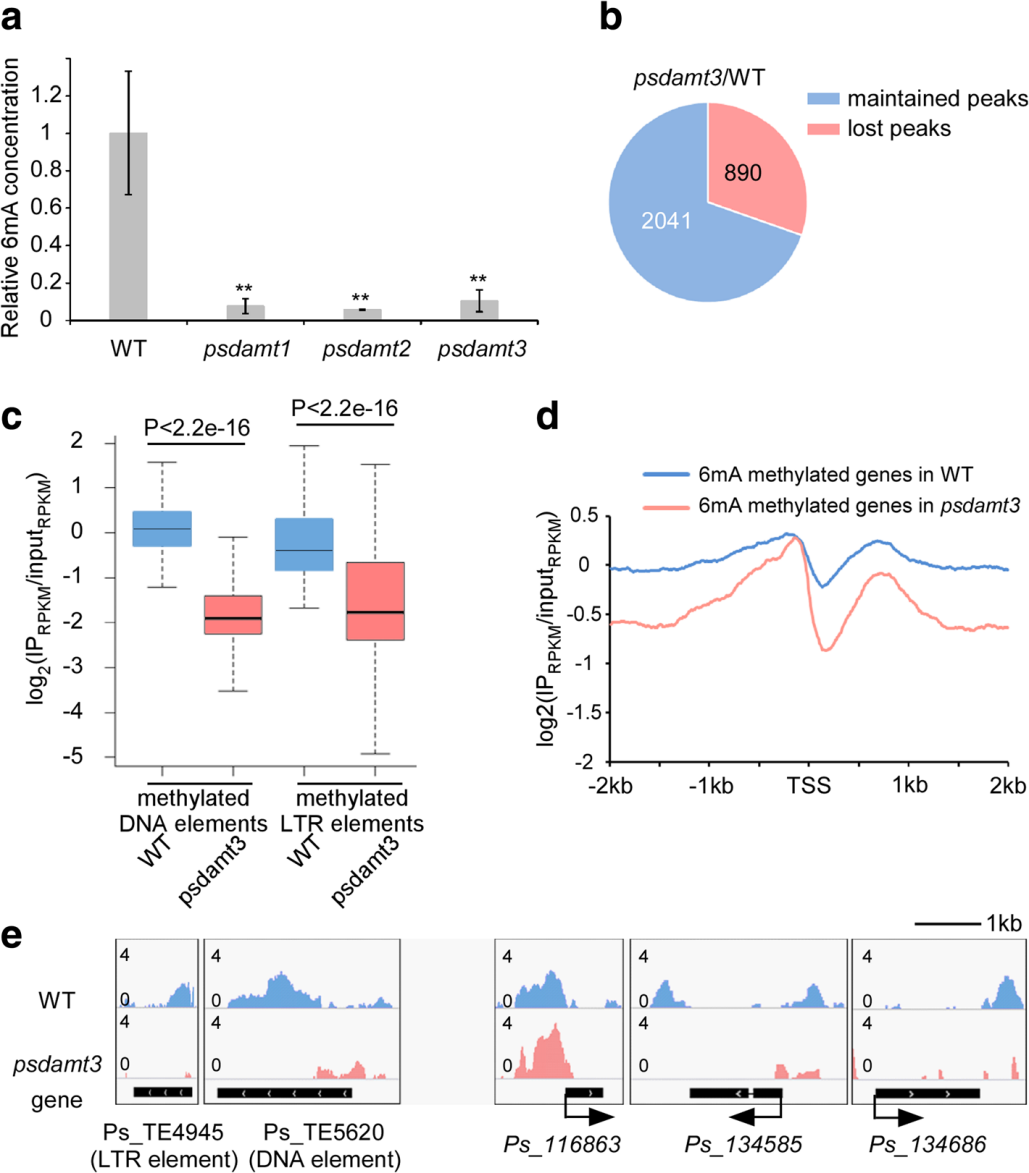
6mA level and landscape are altered in the *Phytophthora* DAMT3 mutant. **a** The 6mA levels of DAMT mutants were quantified by UPLC-ESI-MS/MS. WT is *P*. *sojae* wild-type strain P6497. *psdamt1* (T21), *psdamt2* (T52), and *psdamt3* (T9) are representative lines from each of the *DAMT* gene knockout mutants generated by CRISPR/Cas9. **Significant differences (*P* < 0.01, Student’s *t* test). **b** A total of 890 (29.4%) 6mA peaks are lost in *psdamt3.* **c** Methylation level of methylated DNA elements (*n* = 480) and LTR elements (*n* = 867) are reduced in *psdamt3*. *P* values are calculated with the two-sample Kolmogorov-Smirnov test. **d** The average 6mA level of methylated genes (*n* = 1343) is reduced in the *psdamt3* mutant. **e** Snapshot of 6mA deposition in the *psdamt3* mutant demonstrates that 6mA reduced in representative genomic segment. Here, a methylation peak in *Ps_116863* gene locus is used as an unaffected control

To further investigate the function of DAMTs in *Phytophthora*, we individually knocked out *DAMT* genes in the *P. sojae* strain P6497 using CRISPR/Cas9 gene editing methodology. We designed two sgRNAs matching two sites in each of the *DAMT* genes and harvested at least three independent knockout transformants for each gene (Additional file 4: Table S5, Additional file 2: Figure S10). We selected homozygous mutants *psdamt1*-T21 (− 139 bp), *psdamt2*-T52 (− 1 bp), and *psdamt3*-T9 (− 374 bp) as representative strains for further analyses. To examine the 6mA level in the *PsDAMT* mutants, we applied UPLC-ESI-MS/MS to quantify 6mA abundance. 6mA levels in *psdamt1*-T21 and *psdamt2*-T52 were dramatically reduced to only 8.1% and 5.8% of the wild-type strain, respectively, whereas *psdamt3*-T9 decreased to 11.0% (Fig. 5a, Additional file 4: Table S6). Although our biochemistry assays showed that PsDAMTs have different levels of enzymatic activities, these experiments show that each of the three *PsDAMT* genes significantly contributes to 6mA modification in vivo. Remarkably, the three enzymes do not appear to be fully redundant as each of the single knockout mutants had a reduced methylation level.

Given that *DAMT3* encodes a functional methyltransferase that is conserved among all examined oomycete species, we examined the methylome in the *psdamt3* T9 mutant in more detail using MeDIP-seq. Eight hundred ninety methylated peaks (29.3%) were lost in *psdamt3* with 2041 peaks still maintained (Fig. 5b). Considering 6mA genomic distribution pattern, we further checked 6mA level changes at TE locus and TSS vicinity region. 6mA signals were weaker in *psdamt3* than wild-type at DNA element and LTR element regions (Fig. 5c, Additional file 2: Figure S11a). Moreover, we observed a global reduction in 6mA levels around TSS (Additional file 2: Figure S11b). The reduction occurred at gene locus from both GSR and GDR across the genome (Additional file 2: Figure S11c). We conclude that the *PsDAMT3-*regulated 6mA methylome is not specifically associated with the bipartite genome architecture. However, a closer examination of the bimodal methylation pattern around the TSS of 6mA methylated genes uncovered a greater loss in the second peak in the *psdamt3* mutant compared to the wild-type (Fig. 5d). This unexpected finding indicates that *DAMT* genes may have some degree of functional specialization and that PsDAMT3 may have a preference for the methylation of gene bodies after the TSS. Representative genomic segments with typical changes in 6mA localization are illustrated for *psdamt3* and wild-type *P. sojae* (Fig. 5e). The MeDIP-seq data partially explains the significant reduction of total 6mA levels in the *psdamt3* mutant, but also illustrates the uneven reduction pattern around the TSS, suggesting that there are complex patterns of 6mA methylation by the expanded 6mA methyltransferases of *P. sojae*.

**Fig. 6:**
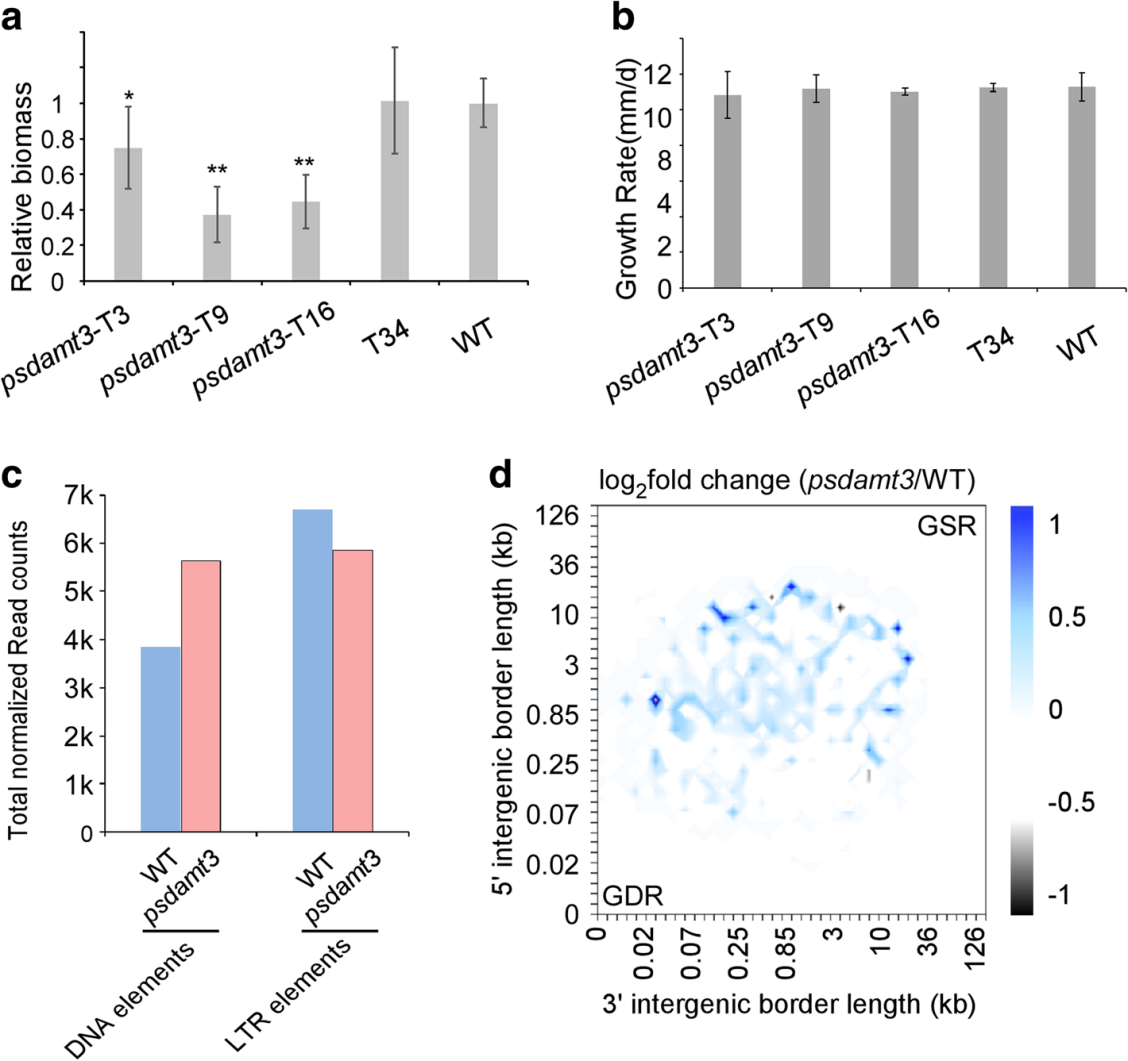
*PsDAMT3* contribute to *P*. *sojae* virulence. **a** Virulence reduced in *psdamt3* mutants. *psdamt3*-T3, *psdamt3*-T9, and *psdamt3*-T16 are *psdamt3* individual mutants, T34 is the transformed strain but DNA sequence did not change, and WT is *P*. *sojae* wild-type strain P6497. The relative *Phytophthora* biomass of the infection tissues was compared with WT. *Significant differences (*P* < 0.05, Student’s *t* test). **Significant differences (*P* < 0.01, Student’s *t* test). **b** In vitro growth rate assay showed *psdamt3* mutants have no growth defects. **c** Total normalized read counts data demonstrated an increase of DNA elements in mutant. **d** Heatmap analyses of transcriptome changes in *psdamt3* compared with wild-type. Gradient color represents the average log_2_fold change of genes in each bin

The genome-wide 6mA reduction in *psdamt3* mutant led us to examine the biological phenotype of the mutant. We therefore examined both virulence and mycelium growth rate of the mutants. Zoospore inoculation assay on soybean-etiolated hypocotyl indicated all three *psdamt3* individual mutants were less virulent than control strains (Additional file 2: Figure S12a). This observation can be also validated by biomass quantification by qRT-PCR (Fig. 6a). To rule out the possibility that reduced virulence was due to defective growth rate, we performed mycelium growth assay and found all the mutants behave normally on media (Fig. 6b, Additional file 2: Figure S12b). Due to the technical limitation of limited pathogen biomass, we currently cannot obtain reliable MeDIP-seq from early infection samples. Therefore, RNA-seq assay on *psdamt3* mutant at mycelium stage was conducted and compared with MeDIP-seq data from mycelium. We firstly examined the expression of methylated gene and TEs. Compared with wide-type strain, the total read counts from DNA elements in *psdamt3* increased by 46.2%, suggesting that DNA elements tend to be more active in *psdamt3* (Fig. 6c, Additional file 4: Table S7), whereas read counts decreased by 12.5% at LTR elements (Fig. 6c, Additional file 4: Table S7). Examination of differentially expressed genes (DEGs) in *psdamt3* uncovers a total of 3156 genes, with 1544 genes upregulated and 1622 genes downregulated (Additional file 2: Figure S13a). Given that only 31 genes among 330 6mA reduced genes were differentially expressed in the *psdamt3* background, we cannot conclude that 6 mA directly represses gene expression. However, mapping DEGs and their expression changes onto gene density heatmaps revealed that larger gene expression variations occur in GSR in the *psdamt3* background (Fig. 6d). Similarly, the length of 5′- and 3′-flanking intergenic region from DEGs was significantly longer than those from core genes and GDR genes (Additional file 2: Figure S14). The data indicated that expression of genes in repeat-rich compartments tend to be more affected in 6mA mutant.

GO analysis of the DEGs revealed that the enriched gene groups are associated with pathogenesis, enzyme activity, response to stimulus, and metabolic and biosynthetic processes, indicating *PsDAMT3* may have a broad regulatory function (Additional file 2: Figure S13b). *PsLAC4*, a known laccase that is required for *P. sojae* pathogenicity [40], from oxidoreductase activity GO category was downregulated (Additional file 2: Figure S13a). Furthermore, the RXLR-type effectors *PsAvr3c* and *PsAvh23*, which are required for *Phytophthora* virulence, were also downregulated in the mutant [41, 42]. Meanwhile, a silenced necrosis-inducing protein *PsNLP40* was activated in *psdamt3* [43], and hypersensitive response-inducing genes *SOJ2C* and *SOJ3D* were also upregulated [44, 45]. This data suggests that *psdamt3* mutant may either reduce the capability to infect the host or trigger stronger plant immune response and partially explains the impaired virulence phenotype of p*sdamt3.*

## Discussion

In this study, we provide evidence that 6mA is a common DNA modification in *Phytophthora* genomes and that this modification may play an important role in the biology and pathology of these eukaryotic microbes. Although 6mA appears to be prevalent in eukaryote genomes, most studies report low levels of abundance. Previous studies documented significant variation of 6mA abundance (6mA/A) ranging from 0.00019 to 2.8% among different eukaryotes [12]. Here, we determined the abundance of 6mA to be 0.05% and 0.04% in the mycelium stage of *P. infestans* and *P. sojae,* respectively. 6mA genome distribution pattern is another way to evaluate its biological significance. Here, we conducted MeDIP-seq, a reliable and widely accepted method in the 6mA research field. Besides MeDIP-seq, 6mA-CLP, 6mA-RE-seq, and SMRT-seq are also used to draw 6mA methylome [12, 15]. Although technical limitation for each method exists, the results from these different methods are consistent to a large extent [15]. *Phytophthora* 6mA methylome generated by other methods will be conducted in the future to obtain an unbiased conclusion of methylation. Unlike reports from some other organisms, 6mA is not evenly distributed across *Phytophthora* genome from our research. We revealed an unexpected link between 6mA methylation and the two-speed genome architecture of *Phytophthora* genomes. We noted an enrichment of 6mA peaks in the intergenic regions, particularly in repeat sequences such as DNA and LTR transposable elements. Other recent studies have suggested that 6mA participates in the regulation of transposon expression in *Drosophila* and mammals [14, 16]. It is recognized that proliferation of TEs could drive the adaptive genome evolution of filamentous pathogens, such as plant pathogenic fungi and oomycetes. To control the activity and the spread of these repetitive sequences, TE-rich regions are normally condensed with a high level of DNA methylation [46, 47]. The genomes of *Phytophthora* species have a greater proportion of repetitive sequences compared to other oomycete species that have been sequenced to date [25]. We speculate that *DAMT* gene expansion in *Phytophthora* species was likely a consequence of transposable-element activity and that the occurrence of 6mA in TE might function to inhibit the activity and spread of TEs in *Phytophthora* species. Indeed, we found the total read counts from DNA elements upregulate by 46.2% in *psdamt3* mutant, suggesting parts of the TEs were becoming more active in line with reduction of methylation level. Therefore, 6mA may play a role in regulating genome integrity and plasticity to the optimal levels necessary for rapid evolution.

Recent studies documented that 6mA is associated with active genes in several organisms, and a popular model is that 6mA may associate with DNA/nucleosome structure to alter gene transcriptional processes [12, 15]. In *Chlamydomonas*, 6mA shows a bimodal localization pattern around TSS and frequently modifies DNA linkers between adjacent nucleosomes around TSS [15]. Studies in *Xenopus laevis* and *Mus musculus* found a marked decrease in 6mA in the vicinity of TSS. 6mA is also predominantly distributed around TSS in a few fungal species [12]. In *P*. *infestans* and *P. sojae*, we observed a bimodal distribution pattern of 6mA-enriched regions flanking the TSS but with a clear depletion at the TSS itself and immediately downstream. This pattern resembles 6mA methylation described for the green algae *Chlamydomonas*. Our comparisons of transcriptome and methylome data suggest that 6mA is associated with lowly expressed gene in the two *Phytophthora* species. Indeed, 6mA depletion is primarily located upstream of TSS in *Chlamydomonas* whereas it is mainly located downstream of TSS in the two *Phytophthora* species. This could account for the apparent different associations of 6mA with gene expression in oomycetes and green algae. Our results are more reminiscent of a recent report in mammalian systems that 6mA is a negative gene expression mark in mouse embryonic stem cells [16]. This is consistent with our hypothesis that 6mA inhibits transposon activity, which is supported by the TE activation observed in *psdamt3* mutant. However, a strong correlation between methylation-changed genes and methylation-induced genes was not observed in *psdamt3* mutant. Thus, we could not simply conclude as to whether 6mA is a repressive mark. A mutant with all three *DAMTs* knocked out will be helpful to further examine gene expression function. Although this correlation is negative, 6mA may interplay with other chromatin remodeling or transcriptional regulators, or maintain the organization of adaptive genomic compartments and as such indirectly regulate gene expression. It is interesting that two *Phytophthora* species do not have detectable 5mC. It is possible that 6mA evolved in *Phytophthora* to be the major DNA methylation players to partially complement the absence of 5mC in this organism. Further investigations are required to explore the roles of 6mA in the modulation of gene expression.

*Phytophthora* genomes are well known for their bipartite “two-speed” architecture with hundreds of gene-sparse regions comprised of repeat sequences and virulence effector genes serving as a cradle for adaptive evolution [27–29]. *Phytophthora* genes in the GSR tend to have higher 6mA levels and are also enriched in plant-induced genes that are normally silenced in vitro [27, 48]. Effector gene silencing has been linked to rapid evolution in both *P. sojae* and *P. infestans* [49, 50]. In addition, *Phytophthora* species tend to exhibit high levels of expression polymorphisms in genes located in the GSR [51]. Indeed, we observed higher 6mA levels in secretome genes and RxLR effector genes (Additional file 2: Figure S9b, c). This data is also consistent with the observation that 6mA marks are associated with low gene expression levels and may, therefore, contribute to the downregulation of virulence effector genes during vegetative stages, as proposed for the fungal pathogen *Leptosphaeria maculans* [52]. The bioinformatics analyses of *psdamt3* mutant revealed that 6mA level was reduced in both GSR and GDR. Whether other two *DAMTs* specifically target to different genomic compartment is an appealing question and deserves further investigation. One interesting observation is that TEs like DNA element tend to be more active in the *psdamt3* mutant. This may imply that the structures or the organizations of GSR that were enriched in TEs were changed. Consistently, we observed that the expression changes of GSR genes were more evident in *psdamt3* mutant, and DEGs in *psdamt3* mutant have longer flanking intergenic region (Additional file 2: Figure S13), suggesting many genes from adaptive genomic regions were indirectly influenced. In summary, we hypothesize that 6mA is involved in TE expression and genomic status changes, thus shaping host adaptation and enhancing evolvability in the plant pathogen *Phytophthora*.

Herein, we identified genes predicted to encode 6mA methyltransferases and demethylases in *P. infestans* and *P. sojae*. We initially focused on studying the functionality of the predicted 6mA methyltransferases to provide evidence that *Phytophthora* species have the inherent capability to perform this DNA modification. Our present work shows that DAMT homologs are the major N6-adenine methyltransferases in *Phytophthora* species. Although MT-A70 homologous proteins function in performing 6mA methylation in *C. elegans* [13], our findings suggest that MT-A70 type methylases do not participate in 6mA methylation in *P. infestans* or *P. sojae*. MT-A70 homologs are either missing or pseudogenized in the *Phytophthora* species we examined. Our results also indicate that DAMTs underwent gene expansion in *Phytophthora* species compared to related oomycete genera such as *Hyaloperonospora* and *Albugo*. Among the three putative methyltransferases we characterized, DAMT3 appears to be the ancestral gene. To our surprise, knockout of each of the *DAMT* genes in *P. sojae* resulted in a substantial and comparable reduction of 6mA abundance in vivo. Like the *psdamt3* mutant, 6mA abundance in the *psdamt1* and *psdamt2* mutants was reduced to a similar level, despite the differences we observed in the in vitro activity of each of the DAMT enzymes. The large scale of DEGs and disordered TEs indicate that *PsDAMT3* has important roles in gene expression. The results suggest that all three *DAMT* genes are required for efficient 6mA methylation and gene expression in *P. sojae*.

The observation that all three *Phytophthora DAMT* genes contribute to 6mA genome methylation is intriguing. Our MeDIP-seq data uncovered that altered 6mA signals from the *psdamt3* mutant are unevenly spread across the genome (Fig. 5). This observation suggests that certain 6mA sites could be preferentially regulated by *PsDAMT1* or *PsDAMT2.* Meanwhile, it also indicates that 6mA gene body modifications after the TSS are preferentially produced by *PsDAMT3*. We propose that gene expansion may have led *Phytophthora* 6mA methyltransferases to specialize; thus, they may not be fully functionally redundant. Although PsDAMT2 showed no detectable methyltransferase activity in the in vitro assay, the *P. infestans* orthologous PiDAMT2 clearly displayed a methyltransferase activity for this enzyme. This indicates that DAMT2 activity may vary between the two *Phytophthora*. PsDAMT2 was not active in vitro, but *DAMT2* knockout mutant had reduced 6mA. These data may appear contradictory. We reason that although in vitro enzyme activity can be measured to some extent, this method is limited and the lack of measurable activity in the in vitro assay does not necessarily mean the enzyme is non-functional. Specifically, the in vitro assay only detects stand-alone methylase activity that modulates GATC sites. Therefore, although enzyme activity could not be detected for PsDAMT2 alone from this assay, we cannot rule out the possibility that PsDAMT2 still functions as an active enzyme. Nevertheless, the mode of action of *Phytophthora* DAMTs in the methylation process and their roles in targeting particular genome compartments require further investigation.

The mechanisms underpinning large-scale gene regulation in *Phytophthora* have remained poorly understood ever since the observations of the internuclear spread of gene silencing by van West and colleagues almost 20 years ago [53]*.* DNA methylation inhibitor 5-azacytidine and histone deacetylase inhibitor trichostatin-A released the silencing state of the *inf1* elicitin gene in *P. infestans*. Silencing Dicer-like, Argonaute, and histone deacetylase genes reversed the expression of sporulation gene *cdc14* [54]. More recently, naturally occurring gene silencing of an avirulence effector gene in *P. sojae* was associated with the appearance of small RNAs [49]. These data suggest that epigenetic regulation plays a role in virulence and development of *Phytophthora* species. Although DNA methylation is a common type of epigenetic modification in many organisms, the extent to which *Phytophthora* genomes are methylated has remained unclear. van West and his colleagues failed to detect 5mC by bisulfite sequencing in an endogenous locus that is sensitive to DNA methylation inhibitor [53]. Our results not only clarify that 5mC is absent in *Phytophthora* species but also provide evidence that 6mA shapes the adaptive genome in this lineage of organisms. To our best knowledge, this is also the first 6mA methylome report from stramenopiles or heterokonts. This work provides a starting point to further explore 6mA regulation in oomycete organisms, with important implications for plant pathology and management of plant diseases. Our results together with emerging studies in other organisms suggest that 6mA fulfills distinct and perhaps differing roles across the spectrum of eukaryotic organisms.

## Methods

### Phytophthora and plant cultivation

*P. sojae* reference strain P6497 was routinely cultured on solid 10% V8 agar medium at 25 °C in the dark. Non-sporulating hyphae were cultured at 25 °C in the dark using 10% V8 liquid medium for 3 days. *P. infestans* T30-4 strain was routinely cultured on the solid RSA/V8 medium at 18 °C in the dark. Non-sporulating hyphae were cultured at 18 °C in the dark in 10% V8 medium for 6–7 days. Hyphae were collected and immediately frozen in liquid nitrogen. Soybean cultivar Hefeng47 and Williams were used to provide etiolated hypocotyl after growing at 25 °C (16 h) and 22 °C (8 h) for 4 days in the dark.

### Data sampling and phylogenetic analyses

For homologous protein search, we selected 23 sequenced species, including 15 oomycete species and 8 model organisms as shown in Fig. 1a. Their genome sequences were downloaded from EnsemblGenomes (http://ensemblgenomes.org/) and Joint Genome Institute (http://genome.jgi.doe.gov/). N6-adenineMlase (PF10237), MT-A70 (PF05063), DAM (PF05869), DNA_Methylase (PF00145), and MethyltransfD12 (PF02086) from the PFAM database were used to BLAST search homologous enzymes with an *E* value cutoff of 10^−5^ [30, 31]. The phylogenetic and molecular evolutionary analyses were conducted using *MEGA* versin6 [55].

### Dot blot assay

Genomic DNA of *P. sojae* and *P. infestans* were extracted using TIANGEN DNAsecure Plant kit. Different amounts of gDNA were denatured at 95 °C for 5 min and chilled in ice for 10 min. DNA were spotted on HybondTM-N+ membranes. The membrane was allowed to dry at 37 °C for 20 min and then crosslinked using HL-2000 HybriLinker for 5 min. The membrane was blocked in 5% milk PBST for 1 h at room temperature and then incubated with 6mA antibody (sysy202003) in 5% milk PBST overnight at 4 °C. After three 10-min washes with PBST, DNA and membrane were incubated with secondary antibody (ab6721) for 30 min at room temperature. After three 10-min washes with PBST, the membrane was treated with Pierce ECL Western Blotting Substrate (Prod#32106) and detected by Tanon-5200Mutil. One hundred nanograms of input DNA of every sample were loaded on 1% agarose gels, followed by air drying for 5 min and photographed using Clinx GenoSens. Relative 6mA abundance was quantified (integrated signal density_anti-6mA_/integrated signal density_input DNA_) using ImageJ [56] from four replicates.

### HPLC analysis for 5mC

The HPLC separation was performed on a Zorbax SB-C18 column (2.1 mm × 150 mm, 5 mm, Agilent) with a flow rate of 0.8 mL/min at 30 °C. Methanol (with 0.1% formic acid, *v*/*v*, solvent A) and 10 mM potassium phosphate monobasic in water (with 0.1% formic acid, *v*/*v*, solvent B) were employed as mobile phase. A gradient of 3 min 90% B with a flow rate of 0.8 mL/min, 1 min 90% B with a flow rate of 0.8–0.2 mL/min, 11 min 90% B with a flow rate of 0.2 mL/min, 3 min 90% B with a flow rate of 0.2–1.2 mL/min, 10 min 90% B with a flow rate of 1.2 mL/min, and 2 min 90% B with a flow rate of 1.2–0.2 mL/min was used.

### UPLC-ESI-MS/MS analysis for 5mC and 6mA

Analysis of the DNA samples was performed on UPLC-ESI-MS/MS system consisting of a Waters Xevo TQ-S micro mass spectrometer (Waters, Milford, MA, USA) with an electrospray ionization source (ESI) and an Acquity UPLC-I-Class™ System (Waters, Milford, MA, USA). Data acquisition and processing were performed using Masslynx software (version 4.1, Waters, Manchester, UK). The UPLC separation was performed on a reversed-phase column (BEH C18, 2.1 mm × 50 mm, 1.7 μm; Waters) with a flow rate of 0.2 mL/min at 35 °C. FA in water (0.1%, *v*/*v*, solvent A) and FA in methanol (0.1%, *v*/*v*, solvent B) were employed as the mobile phase. A gradient of 5 min 5% B, 10 min 5–30% B, 5 min 30–50% B, 3 min 50–5% B, and 17 min 5% B was used. The mass spectrometry detection was performed under positive electrospray ionization mode. 6mA retention time is around 2.56 min. The mass transition for 6mA was set as *m*/*z* 266.02 > 149.95. The mass transition for dA was set as *m*/*z* 252.04 > 116.94.

A HPLC-ESI-MS/MS system, consisting of an electrospray-time-of-flight mass spectrometry (Triple TOF 5600+, AB Sciex) and liquid chromatography (LC-20ADXR HPLC, Shimadzu), was also used for 5mC detection. Data acquisition and processing were performed using PeakView version 2.0 (AB Sciex). The HPLC separation was performed on a reversed-phase column (C18, 2.1 mm × 100 mm, 2.6 μm; Kinetex) with a flow rate of 0.2 mL/min at 40 °C. FA in water (0.1%, *v*/*v*, solvent A) and FA in methanol (0.1%, *v*/*v*, solvent B) were employed as the mobile phase. A gradient of 15 min 20–90% B, 3 min 90% B, 0.1 min 90–20% B, and 1.9 min 20% B was used. The mass spectrometry detection was performed under ESI positive mode with a DuoSpray dual-ion source. Retention time of 5mC is around 1.79 min. The mass transition for 5mC was set as *m*/*z* 242.11 > 126.05.

### DpnI-dependent methylation assay

DpnI-dependent methylation assay was performed as previously described [35]. The reaction contained 20 mM Tris-HCl, pH 8.0, 50 mM NaCl, 7 mM 2-mercaptoethanol, 1 mM EDTA, 0.1 mg/ml bovine serum albumin (BSA), 1 μg N6-methyladenine-free lambda DNA, purified recombinant proteins (1–27 μg), and 50 μM unlabeled AdoMet. The reaction system was incubated at 37 °C for 1 h and then 65 °C for 15 min to stop the reaction. The methylated DNA was digested by 5 U DpnI at 37 °C for 1 h. Digestion was stopped by heat inactivation by incubating at 80 °C for 20 min. One percent agarose gel electrophoresis was used to check digestion. PsDAMT1, PsDAMT2, PsDAMT3, DAM, and PsAvr3c were cloned into pET32a-c (+). The recombinant plasmids were transformed into *E. coli* HST04 strain (*dam-*, *dcm-*). Bacteria were grown overnight at 37 °C. *E. coli* gDNA were extracted using TIANamp Bacteria DNA Kit. DpnI 1% agarose gel electrophoresis was used to check digestion results. Elution buffer of the proteins contains 10% glycerol which could influence the DNA band shift.

### MeDIP-seq (6mA-IP-seq)

MeDIP-seq used in this paper was optimized from several protocols [13–15]. gDNA was extracted using TIANGEN DNASeure Plant Kit and then treated with RNase A overnight. The gDNA was diluted to 100 ng/μL with TE buffer, and 100 μL diluted gDNA was put in each tube and sonicated to 200–400 bp using Biorupter UCD-600. The 200–400-bp-sized DNA was extracted using Takara Gel DNA Extraction Kit ver.4.0. DNA was denatured at 95 °C for 10 min and chilled in ice immediately for 5 min. Twenty microliters of denatured DNA was stored as input. The rest of the DNA was incubated with 3 μg 6mA antibody at 4 °C for more than 6 h. Dyna beads (Thermofisher 10001D) were washed twice using 1 × IP buffer and pre-blocked in 0.8 mL 1 × IP buffer with 20 μg/μL BSA. Pre-blocked beads were washed twice using 1 × IP buffer (5 × IP buffer: 50 mM Tris-HCl, 750 mM NaCl, and 0.5% vol/vol IGEPAL CA-630), and then, incubated DNA-antibody was added to pre-blocked beads and rotated overnight at 4 °C. The beads were washed four times for 10 min with 1 × IP buffer. IP products were suspended in 400 μL preheated elution buffer at 65 °C for 15 min to yield the 6mA-IPed library; the step was repeated using 300 μL preheated elution buffer (elution buffer: 50 mM NaCl, 20 mM Tris-HCl, 5 mM EDTA, 1% SDS). Eluted DNA was combined and then added to an equal volume of phenol-chloroform-isopentanol, vortexed and centrifuged at 13,000 rpm for 5 min at room temperature. The aqueous phase was transferred into a new tube and mixed with an equal volume of ethanol to precipitate the eluted DNA. The library was prepared using VAHTS™ Turbo DNA Library Prep Kit for Illumina and VAHTS™ Multiplex Oligos set 1 for Illumina and sequenced by BGI (Shenzhen, China) and GENEWIZ (Suzhou, China).

### *Phytophthora* transformation

*Phytophthora* CRISPR/Cas9 gene editing and transformation was performed as previously described [57]. The sgRNA target sites were selected using an online tool (http://grna.ctegd.uga.edu/).

### RNA extraction, RNA-seq, and qRT-PCR

Total RNA of 3-day-old *P. sojae* hyphae were isolated using Omega Total RNA Kit I according to the manufacturer’s manual. RNA quantity and quality was measured using Nanodrop ND-1000 and 1% agarose gel electrophoresis. RNA-seq service was provided by BGI and 1gene. RNA reverse transcription was conducted using Takara PrimerScript™ RT reagent kit with gDNA eraser. Quantitative RT-PCR was performed using the ABI PRISM 7500 Fast Real-Time PCR System.

### High-throughput sequence data analysis

RNA-seq data was mapped to *P. sojae* v1.1 using Tophat2, and MeDIP-seq data was mapped to *P. sojae* v1.1 using bowtie2. Gene expression data were generated by Cufflinks. MeDIP-seq data was normalized and visualized using deepTools [58] and IGV [59]. 6mA methylation peaks were called using SICER. The figure of two-speed genome was produced as described before [60]; 6mA distribution was calculated as log_2_(IP_RPKM_/input_RPKM_ + 1), RPKM value from the regions before TSS 500 bp (real length and reads number calculated when the length of flanking intergenic regions < 500 bp, because the flanking intergenic regions between a few genes are shorter than 500 bp). *Phytophthora* repeat sequences were referenced in previous publications [25] and re-annotated here by RepeatMasker [61]. Read counts were calculated using featureCounts, and DEGs were calculated using DEseq2 [62] with |log_2_FC| ≥ 1; *y*-axis is FDR < 0.05. GO annotation was performed using Gostats [63].

### Virulence assay

Soybean cultivar Hefeng47 was used to provide etiolated hypocotyl after growing at 25 °C (16 h) and 22 °C (8 h) for 4 days in the dark. Hypocotyls of etiolated soybean seeding were incubated using 100 zoospores. Photos of infection were taken at 48hpi. Relative virulence was quantified by qRT-PCR.

## Additional files

Additional file 1: **Table S1.** HMMER searching result of 5mC methyltransferase in 15 oomycetes. (XLSX 9 kb)

Additional file 2: Supplementary materials. PDF document with supplementary figures. (PDF 2073 kb)

Additional file 3: **Table S2.** HMMER searching result of DAMT in 15 oomycetes. (XLSX 20 kb)

Additional file 4 Supplementary materials. PDF document with supplementary tables. (PDF 24 kb)

Additional file 5: **Tables S4–S9.** GO analysis result of methylated genes in two *Phytophthora*. (XLSX 59 kb)

## Abbreviations

5mC: 5-Methylcytosine
6mA: Adenine N6-methylation
AlkB: Alkylation repair homologs
DAMT: N6-adenineMlase domain-containing
ESI: Electrospray ionization source
FPKM: Fragments per kilobase million
GDR: Gene-dense regions
GO: Gene ontology
GSR: Gene-sparse and repeat-rich regions
HPLC: High-performance liquid chromatography
LTR: Long terminal repeat
METTL14: Methyltransferase-like protein 14
METTL3: Methyltransferase-like protein 3
PPB: Parts per billion
SMRT-seq: Single-molecule real-time sequencing
