# Supplementary material for: Phytophthora methylomes are modulated by 6mA methyltransferases and associated with adaptive genome regions
Source: Genome Biol. 2018 Oct 31;19:181. doi: 10.1186/s13059-018-1564-4 (PMC6211444; doi:10.1186/s13059-018-1564-4)
Supplement: Supplementary file 2 — Supplementary materials. PDF document with supplementary figures. (PDF 2073 kb) [file 13059_2018_1564_MOESM2_ESM.pdf]

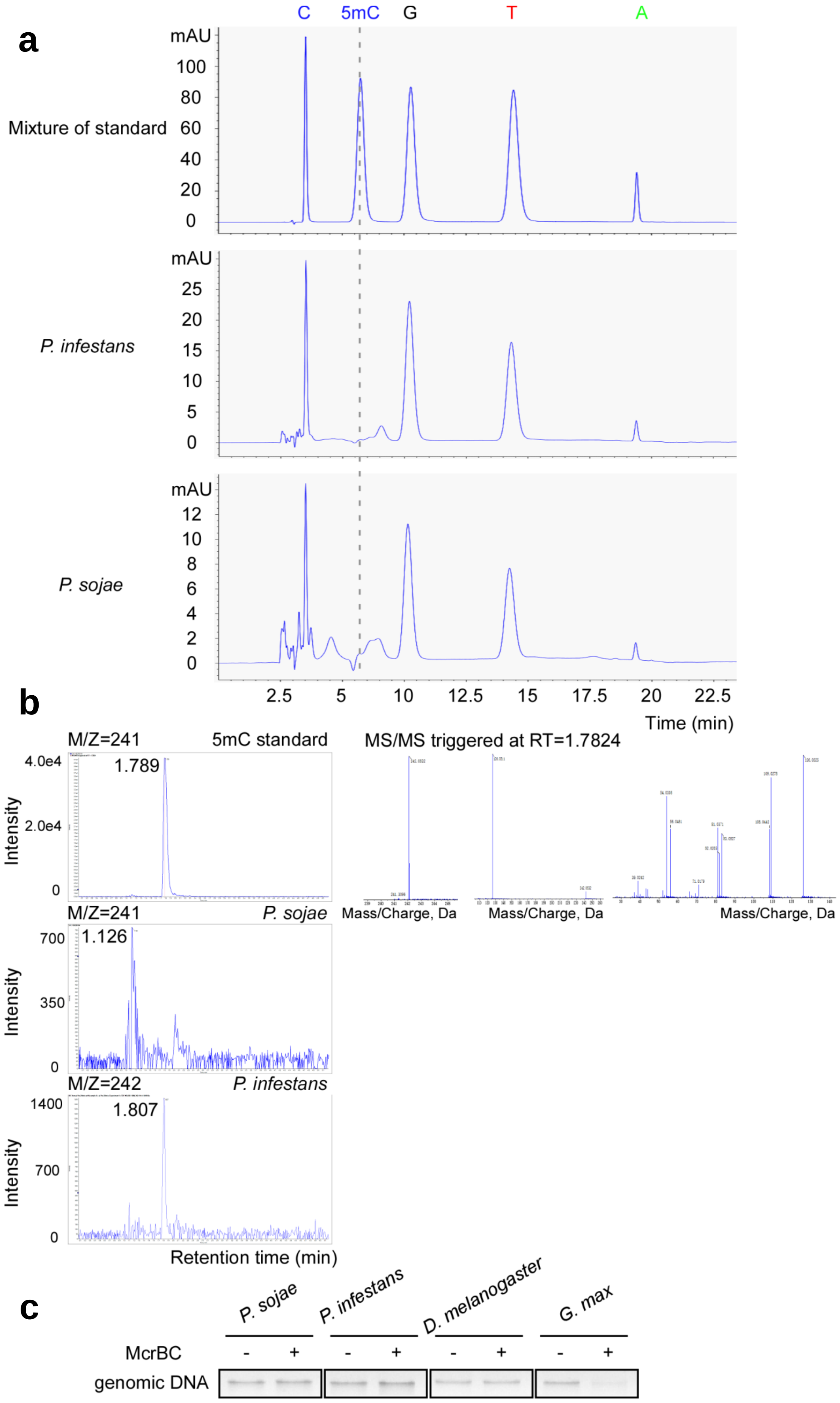

a
C
5mC
G
T
A
mAU
100
80
60
40
20
0
Mixture of standard
mAU
25
20
15
10
5
0
P. infestans
mAU
12
10
8
6
4
2
0
P. sojae
2.5
5
10
12.5
15
17.5
20
22.5
Time (min)
b
M/Z=241
5mC standard
MS/MS triggered at RT=1.7824
4.0e4
2.0e4
0
1.789
Intensity
Mass/Charge, Da
Mass/Charge, Da
Mass/Charge, Da
M/Z=241
P. sojae
700
350
0
1.126
Intensity
M/Z=242
P. infestans
1400
700
0
1.807
Intensity
Retention time (min)
c
P. sojae
P. infestans
D. melanogaster
G. max
McrBC
- + - + - + - +
genomic DNA

Figure S1
No detectable 5mC in *P. infestans* and *P. sojae*
(a) Chromatograms from a standard mixture and from samples of hydrolyzed gDNA from *P. infestans* and *P. sojae.* Samples were separated using HPLC with UV detection. The bases A, T, C, G, were detected in all samples, whereas the modified bases 5mC and 6mA occur only in the standard mixture, as shown.
(b) Results from a UPLC-ESI-MS/MS analysis of a control sample of 5mC, and corresponding chromatographic regions from hydrolyzed gDNA from *P. sojae* and *P. infestans,* showing that 5mC cannot be detected in the test samples.
(c) Genomic DNA samples from *P. sojae, P. infestans, D. melanogaster*, and *G. max* were digested with the 5mC methylation dependent restriction enzyme McrBC. *D. melanogaster* and *G. max* were used as controls. “-” means didn’t add McrBC, “+” means added McrBC.
All the experiments were independently performed with a minimum of three biological replicates, with comparable results.

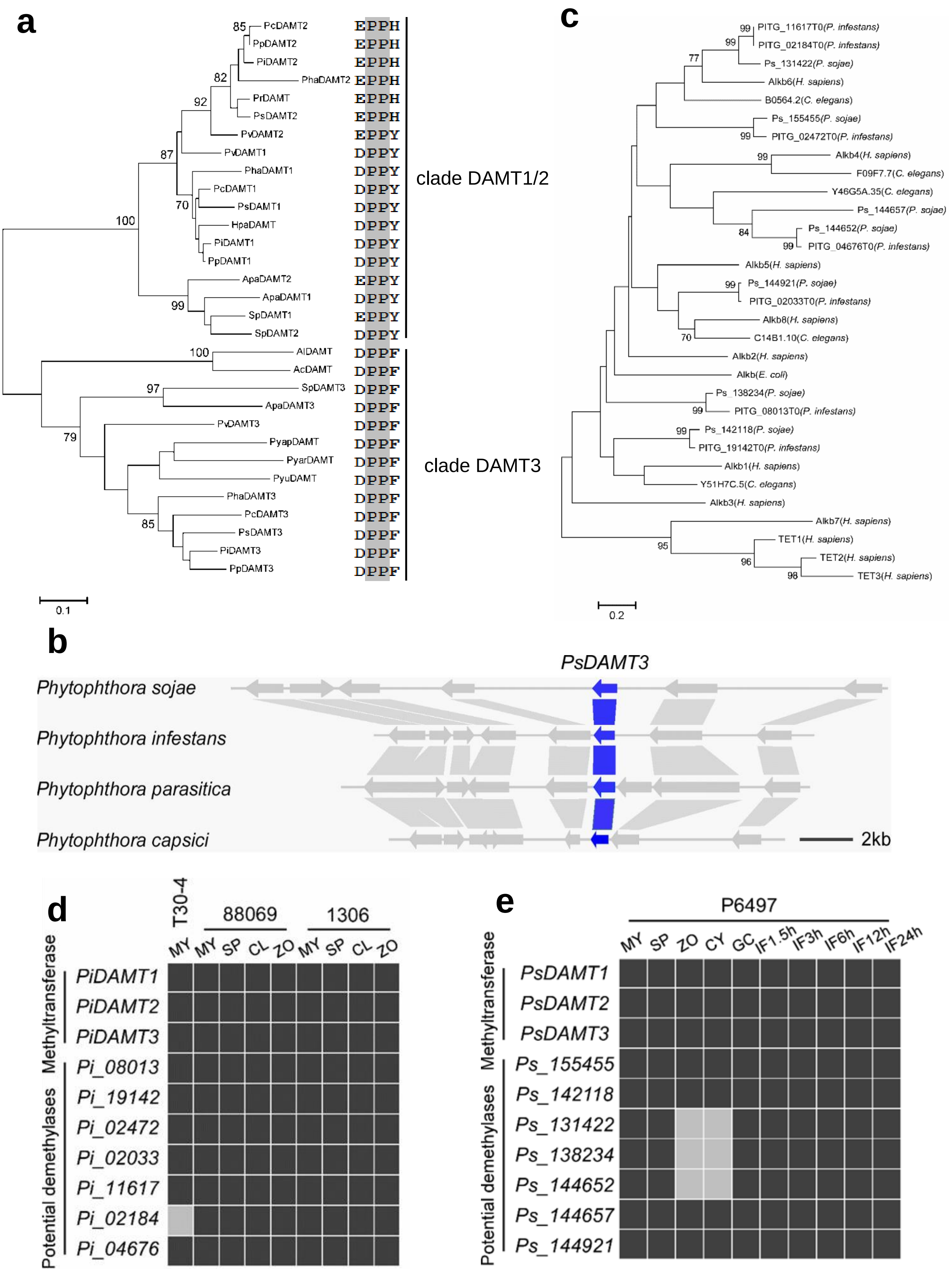

a
PcDAMT2
PpDAMT2
PiDAMT2
PhaDAMT2
PrDAMT
PsDAMT2
PvDAMT2
PvDAMT1
PhaDAMT1
PcDAMT1
PsDAMT1
HpaDAMT
PiDAMT1
PpDAMT1
ApaDAMT2
ApaDAMT1
SpDAMT1
SpDAMT2
AlDAMT
AcDAMT
SpDAMT3
ApaDAMT3
PvDAMT3
PyapDAMT
PyarDAMT
PyuDAMT
PhaDAMT3
PcDAMT3
PsDAMT3
PiDAMT3
PpDAMT3
EPPH
DPPY
EPPY
DPPF
clade DAMT1/2
clade DAMT3
0.1
c
PITG_11617T0(P. infestans)
PITG_02184T0(P. infestans)
Ps_131422(P. sojae)
Alkb6(H. sapiens)
B0564.2(C. elegans)
Ps_155455(P. sojae)
PITG_02472T0(P. infestans)
Alkb4(H. sapiens)
F09F7.7(C. elegans)
Y46G5A.35(C. elegans)
Ps_144657(P. sojae)
Ps_144652(P. sojae)
PITG_04676T0(P. infestans)
Alkb5(H. sapiens)
Ps_144921(P. sojae)
PITG_02033T0(P. infestans)
Alkb8(H. sapiens)
C14B1.10(C. elegans)
Alkb2(H. sapiens)
Alkb(E. coli)
Ps_138234(P. sojae)
PITG_08013T0(P. infestans)
Ps_142118(P. sojae)
PITG_19142T0(P. infestans)
Alkb1(H. sapiens)
Y51H7C.5(C. elegans)
Alkb3(H. sapiens)
Alkb7(H. sapiens)
TET1(H. sapiens)
TET2(H. sapiens)
TET3(H. sapiens)
0.2
b
PsDAMT3
Phytophthora sojae
Phytophthora infestans
Phytophthora parasitica
Phytophthora capsici
2kb
d
T30-4
88069
1306
MY MY SP CL ZO MY SP CL ZO
Methyltransferase
PiDAMT1
PiDAMT2
PiDAMT3
Potential demethylases
Pi_08013
Pi_19142
Pi_02472
Pi_02033
Pi_11617
Pi_02184
Pi_04676
e
P6497
MY SP ZO CY GC IF1.5h IF3h IF6h IF12h IF24h
Methyltransferase
PsDAMT1
PsDAMT2
PsDAMT3
Potential demethylases
Ps_155455
Ps_142118
Ps_131422
Ps_138234
Ps_144652
Ps_144657
Ps_144921

Figure S2

Conservation and gene expression of putative methyltransferases and demethylases in *Phytophthora* species.

(a) Neighbour-Joining tree of N6-adenineMlase domain-containing proteins (DAMTs) in selected oomycete species and their catalytic motifs, shown on the right. More details are in Supplementary Table 2.

(b) Synteny of the *DAMT3* gene clusters in four *Phytophthora* species. Blue arrows are *PsDAMT3* orthologs. Orthologs marked with blue (*DAMT3* orthologs) and grey (others) lines.

(c) Neighbour-Joining tree of potential DNA and RNA demethylase .Bootstrap percentage >70 was shown.

(d) Expression level of three methyltransferases and seven potential demethylases in different developmental stages of the *P. infestans* strains T30-4, 88069, and 1306 from RNA-seq data. Color codes: high expression (FPKM>1, black), low expression or no expression (FPKM<1, grey). 10 stages: MY for mycelium, SP for sporangium, CL for sporangia undergoing zoosporogenesis, ZO for zoospore, CY for cyst.

(e) Expression level of methyltransferases and potential demethylases in 10 developmental stages of the *P. sojae* P6497 strain from RNA-seq data that were published earlier. GC for geminated cysts, IF1.5h-24h for 1.5 hours to 24 hours post infection.

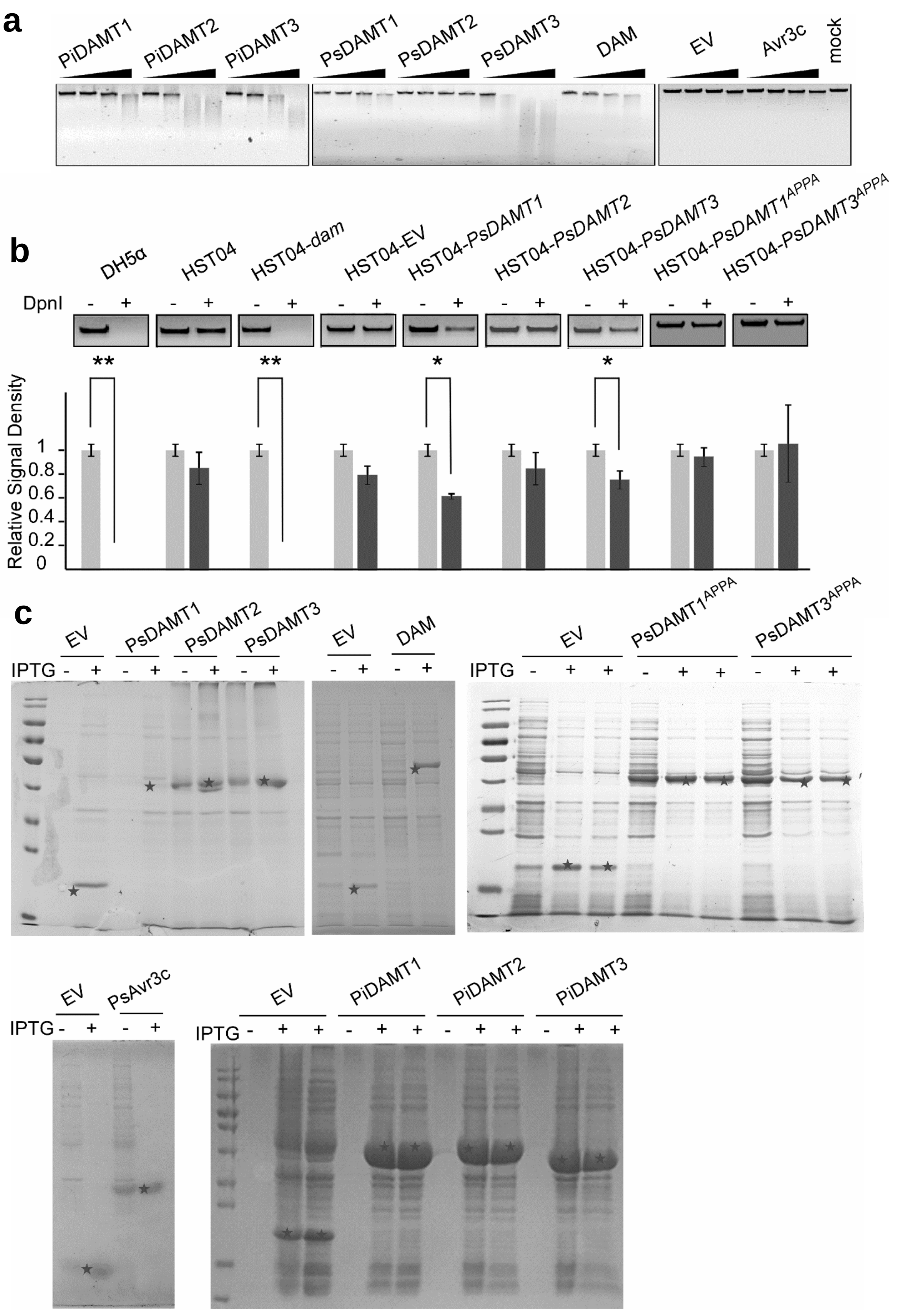
a
PiDAMT1
PiDAMT2
PiDAMT3
PsDAMT1
PsDAMT2
PsDAMT3
DAM
EV
Avr3c
mock
b
DH5α
HST04
HST04-dam
HST04-EV
HST04-PsDAMT1
HST04-PsDAMT2
HST04-PsDAMT3
HST04-PsDAMT1APPA
HST04-PsDAMT3APPA
DpnI
- +
**
*
Relative Signal Density
0
0.2
0.4
0.6
0.8
1
c
EV
PsDAMT1
PsDAMT2
PsDAMT3
DAM
PsDAMT1APPA
PsDAMT3APPA
IPTG
PsAvr3c
PiDAMT1
PiDAMT2
PiDAMT3

Figure S3

In vitro methylation assay of DAMTs, bacteria methyltransferase complementation assay and production of recombinant *P. sojae* methyltransferase

(a) In vitro DpnI-dependent DNA methylation assay suggests that Phytophthora DAMTs have methyltransferase activity. Recombinant proteins PsDAMT1, PsDAMT2, PsDAMT3, PiDAMT1, PiDAMT2, PiDAMT3 together with bacteria 6mA DNA methylase (DAM) were produced in E. coli. EV (empty vector) and Avr3c (a Phytophthora secretion protein) were used as controls. The recombinant protein gradient ranged from 1 μg to 27 μg in each reaction. The experiments were carried out by triplicates with similar results.

(b) DpnI digestion patterns of gDNA extracted from the *E. coli* strains DH5α, HST04, and HST04 containing pET-32a-c (+) with native and mutated genes. Both PsDAMT1^{APPA} and PsDAMT3^{APPA} are mutants with catalytic residues substituted with alanine (A). Signal density of the DpnI digestion image was analyzed using software (ImageJ). * and ** represent significant differences (P<0.05 and P<0.01,Students' t test respectively)

(c) SDS-PAGE analysis of pET32a, pET32a-PsDAMT1, pET32a-PsDAMT2, pET32a-PsDAMT3, pET32a-DAM, pET32a-PsDAMT1^{APPA}, pET32a-PsDAMT3^{APPA}, pET32a-PsAvr3c, pET32a-PiDAMT1, pET32a-PsiAMT2 and pET32a-PiDAMT3 protein expression. Mutagenesis of PsDAMT1 catalytic motif into APPA abolished the enzyme activity. The recombinant protein bands are indicated by the red asterisks."-" means didn't added IPTG, "+" means added IPTG

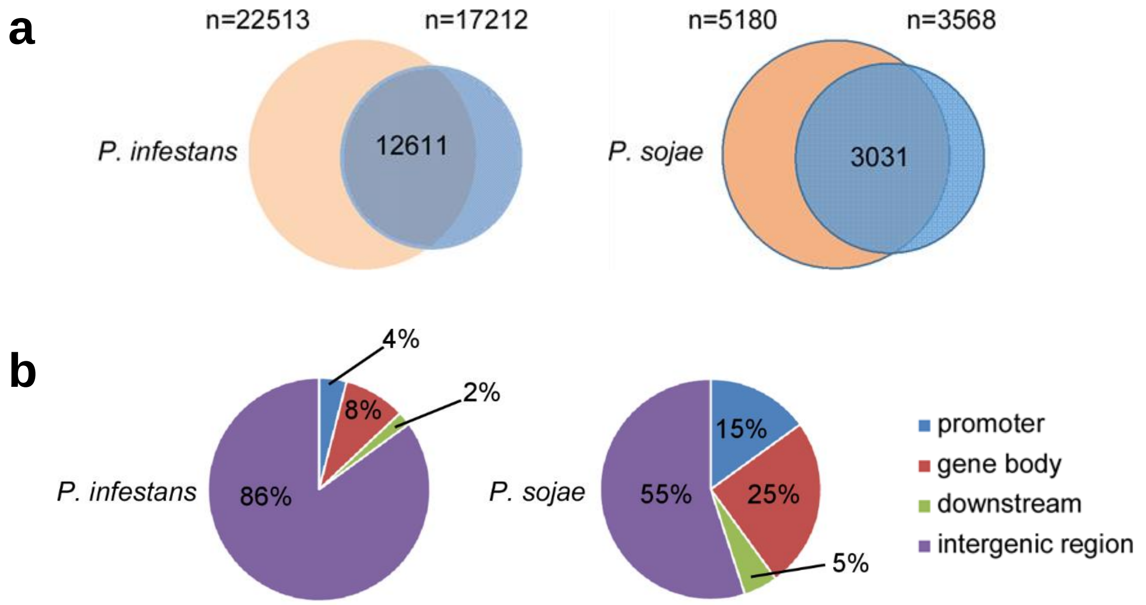

Figure S4
6mA peak identification and distribution across the genomes of *P. infestans* and *P. sojae*
(a) Venn diagram shows number of unique and overlapping 6mA peaks identified from two MeDIP-seq replicates in *P. infestans* and *P. sojae.*
(b) Pie chart of 6mA enrichment peak distribution among gene features in *P. infestans* and *P. sojae.*

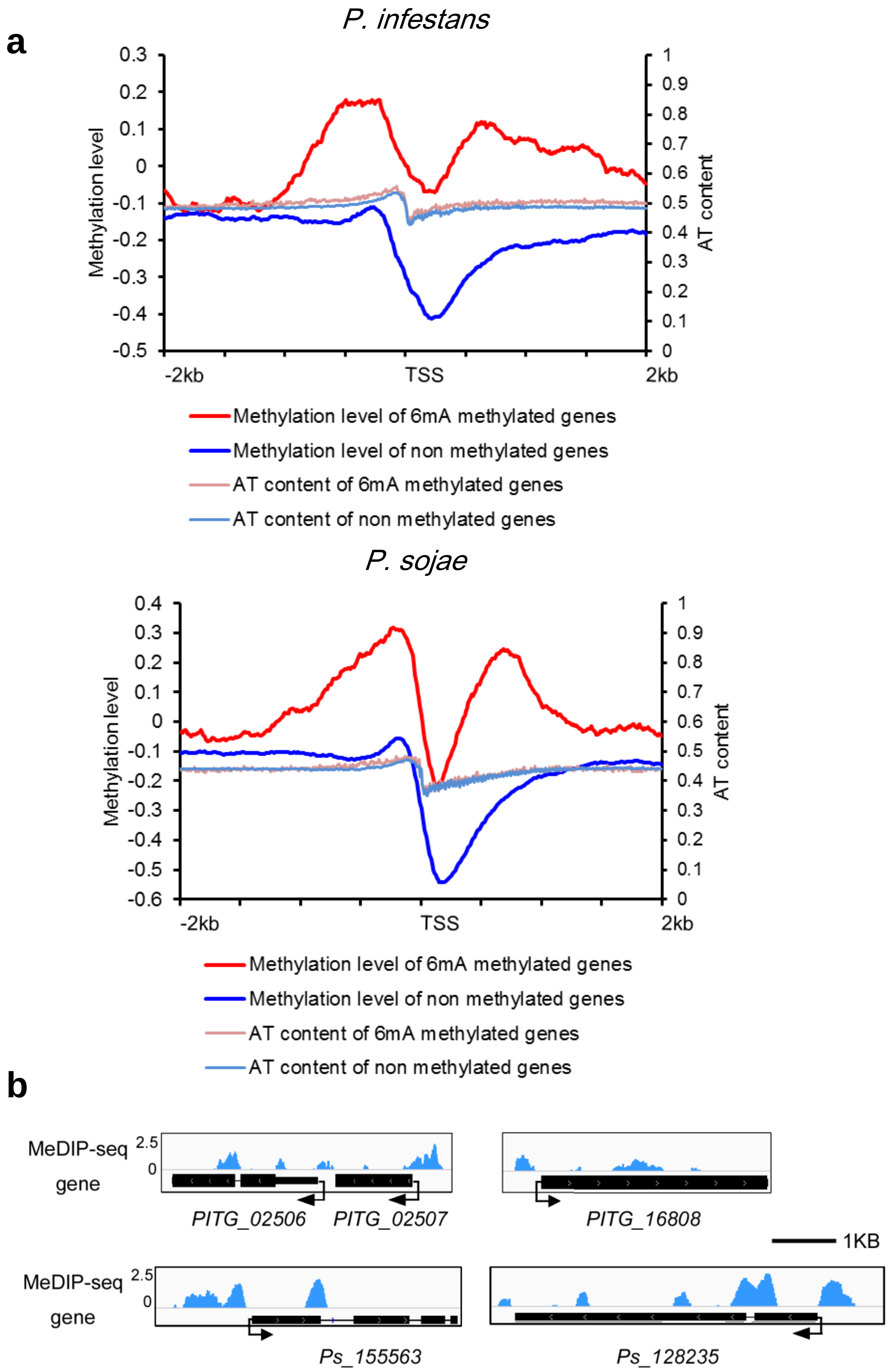
a
P. infestans
Methylation level
AT content
-2kb
TSS
2kb
Methylation level of 6mA methylated genes
Methylation level of non methylated genes
AT content of 6mA methylated genes
AT content of non methylated genes
P. sojae
Methylation level
AT content
-2kb
TSS
2kb
Methylation level of 6mA methylated genes
Methylation level of non methylated genes
AT content of 6mA methylated genes
AT content of non methylated genes
b
MeDIP-seq
gene
PITG_02506
PITG_02507
PITG_16808
1KB
MeDIP-seq
gene
Ps_155563
Ps_128235

Figure S5
Methylated DNA immuno-precipitation sequencing (MeDIP-seq) read deposition in representative loci.
(a) Comparison of methylation level and AT content around TSS -2kb to 2kb. Bin is 10 bases. Left Y-axis is methylation level, calculated as $log_2(RPKM_{IP}/RPKM_{input})$. Right Y-axis is AT content, calculated as (A+T)/(A+T+C+G+N).
(b) Snapshot of 6mA deposition around selected genes in *P. infestans and P. sojae*. Y-axis is $log_2(RPKM_{IP}/RPKM_{input})$.

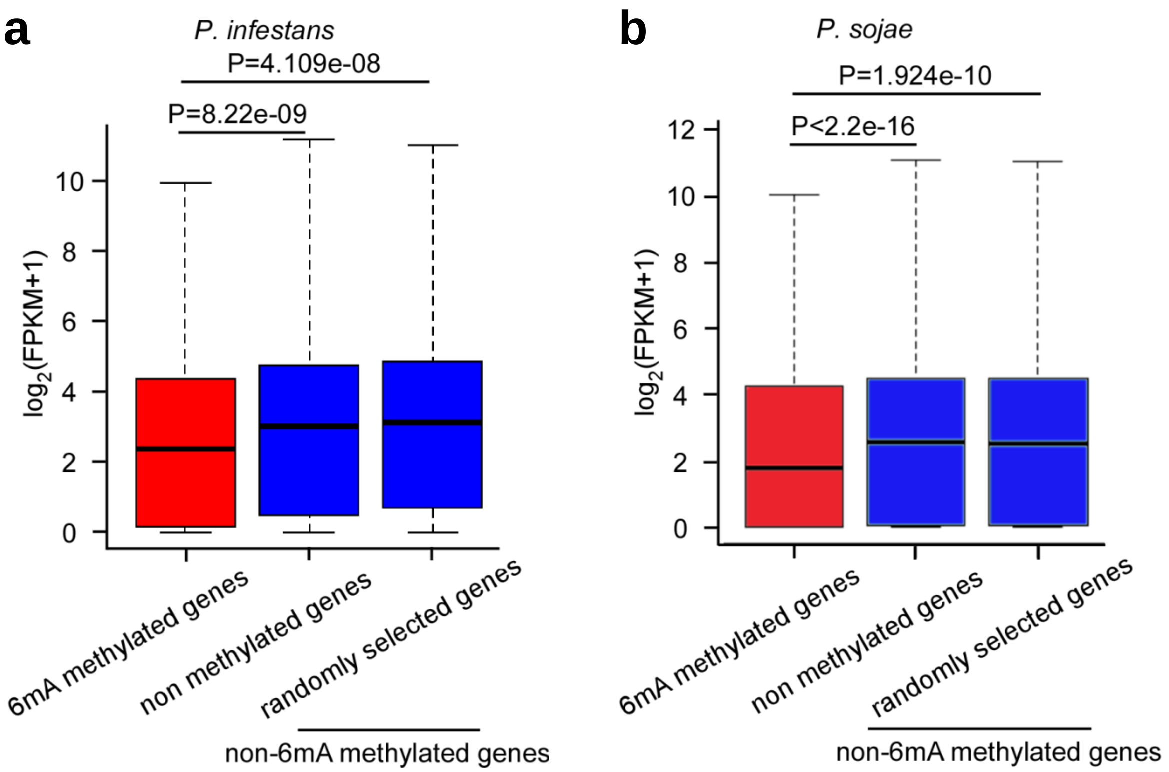

Figure S6

Gene expression levels are negatively associated with 6mA methylation levels

(a) In *P. infestans*, 6mA modified genes (n=1805) (red), randomly selected non-modified genes (n=1805), and all non-modified genes (n=16374) (blue) are shown.

(b) In *P. sojae*, 6mA modified genes (n=1343), randomly selected non-modified genes (n=1343) and all non-modified genes (n=17853) are shown. Different groups of genes are shown along the x-axis. The y-axis represents the relative expression level. P values are calculated with the Two-sample Kolmogorov-Smirnov test.

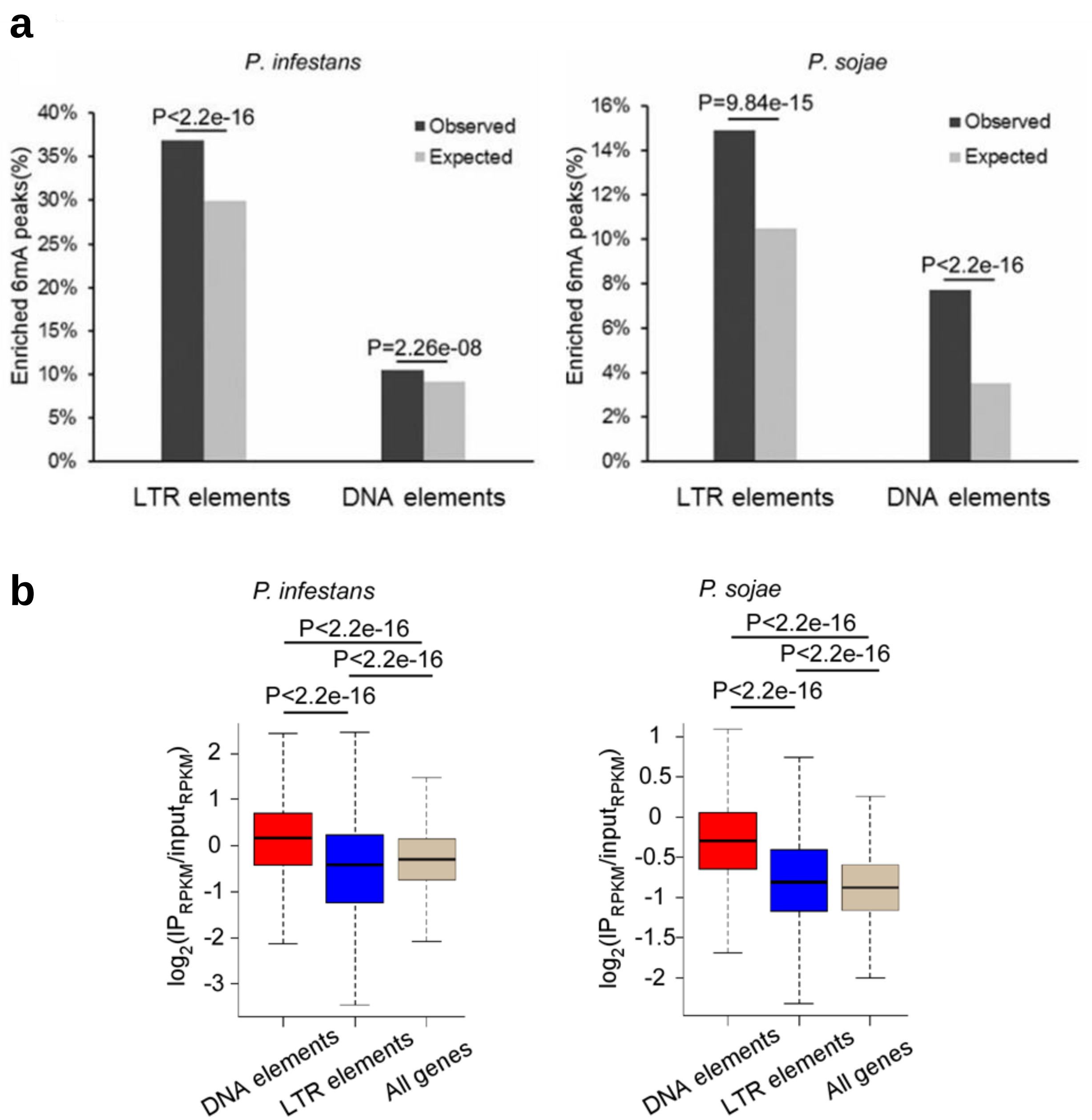

Figure S7

6mA predominantly localized in repetitive and transposable elements

(a) 6mA peaks are enriched in LTR elements and DNA elements. Y-axis is a percentage of enriched 6mA peaks. P values were calculated with excel binomial distribution probability.

(b) DNA elements have a higher methylation level. Y-axis is relative methylation level. P values calculated with the Two-sample Kolmogorov-Smirnov test.

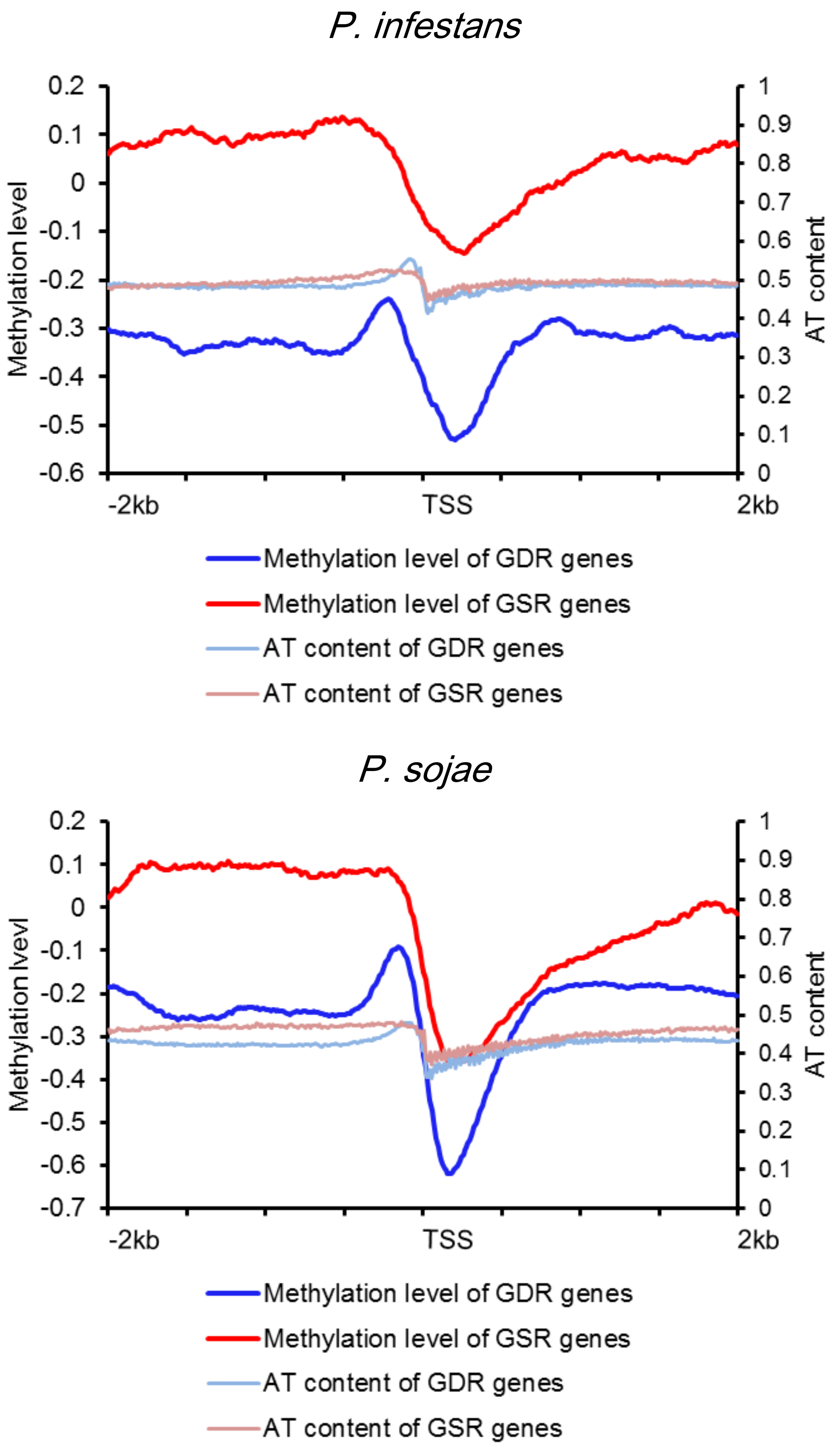

Figure S8

Compression of methylation level and AT content shows no bias in analyzing 6mA enrichment between GDR and GSR genes.

Bin is 10 bases. Left Y-axis is methylation level, calculated as $\log_2(RPKM_{IP}/RPKM_{input})$. Right Y-axis is AT content, calculated as (A+T)/(A+T+C+G+N).

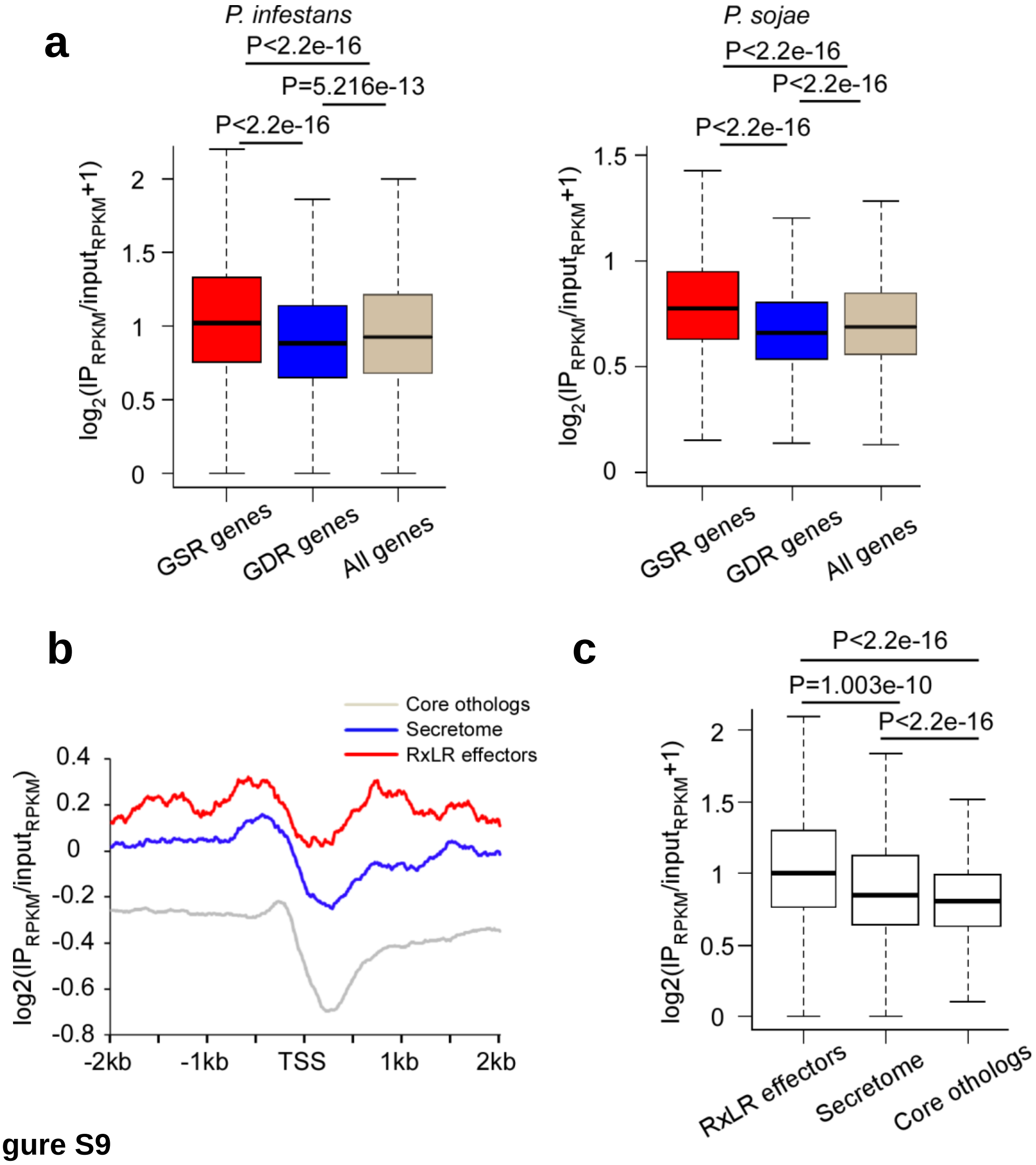

Figure S9

GSR genes, secretome genes and RxLR effector genes have higher 6mA levels

(a) Methylation level of GSR gens is higher than GDR genes and all genes. Y-axis is relative 6mA level. P values are calculated with the Two-sample Kolmogorov-Smirnov test.

(b) The relative 6mA levels of core orthologs (n=7113), secretome genes (n=1415), and RxLR effector genes (n=562) around TSS in *P. infestans* are calculated.

(c) The average relative 6mA levels from core orthologs, secretome genes, and RxLR effector genes in *P. infestans* are shown in a Box blot. P values are calculated with the Two-sample Kolmogorov-Smirnov test.

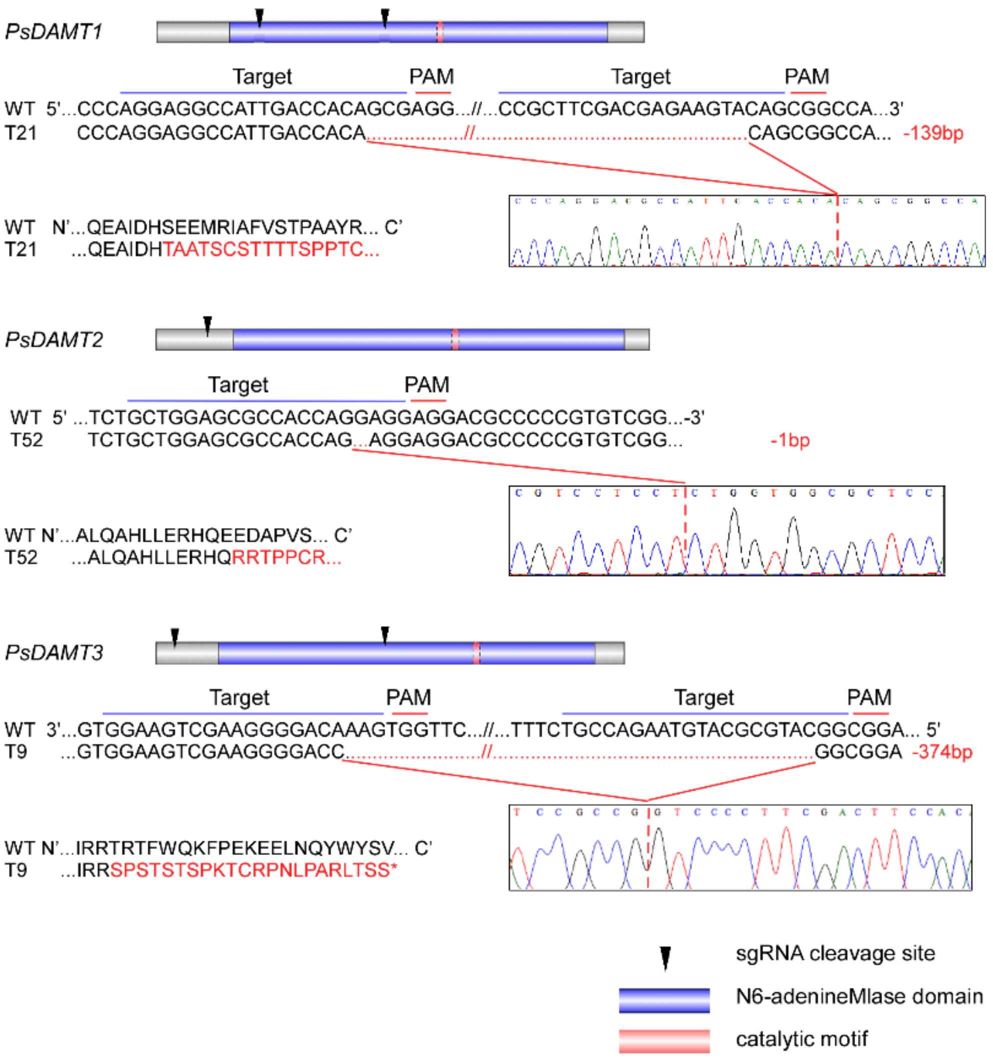

Figure S10

Schematic representations of *PsDAMTs* knockout mutants

Representative mutants *psdamt1* (T21), *psdamt2* (T52), and *psdamt3* (T9) that were generated using CRISPR/Cas9 system are illustrated.

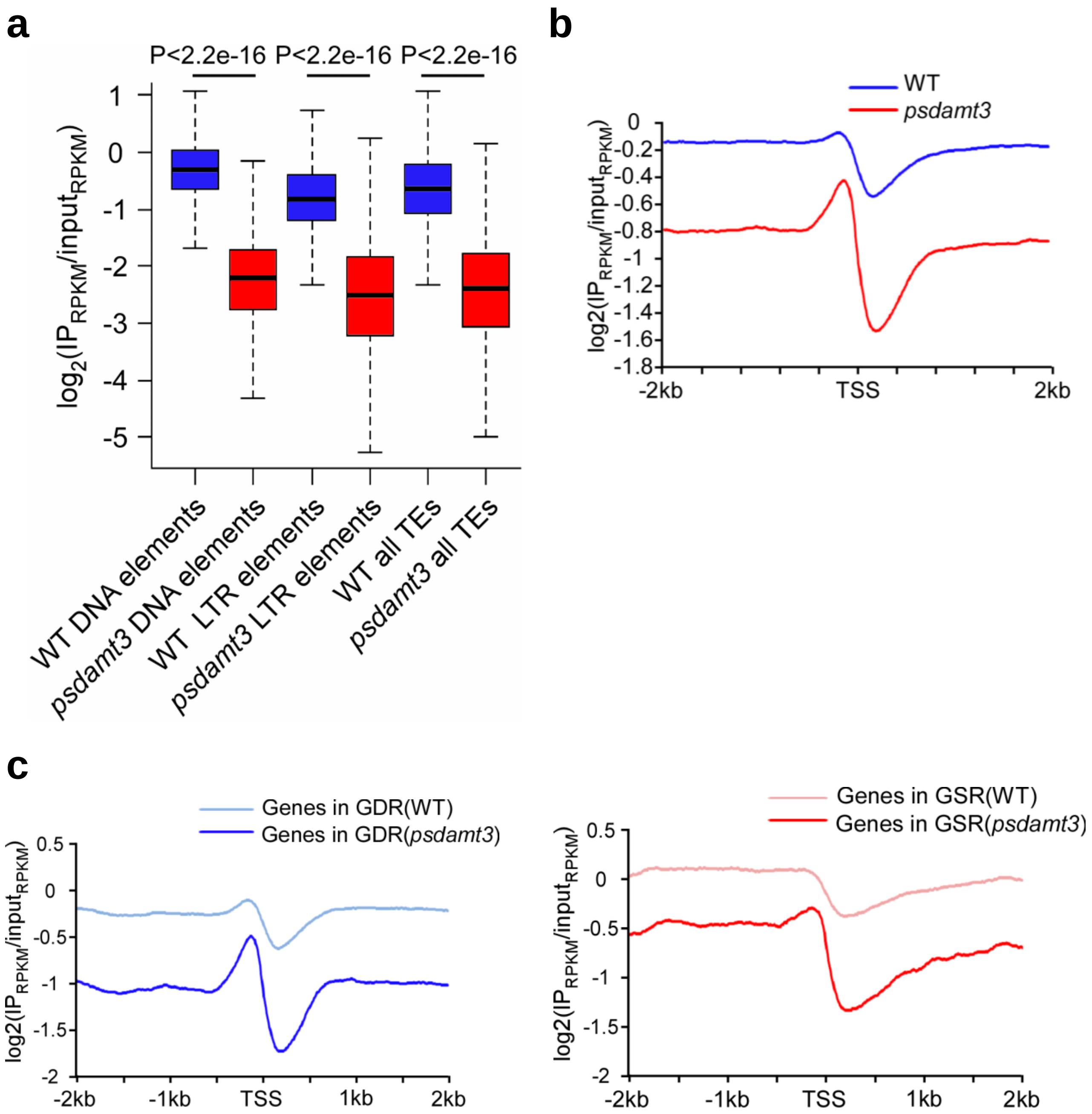

Figure S11
6mA level reduced in TEs and genomic regions
(a) Methylation level of methylated DNA elements (n=3684) and LTR elements (n=8234) are reduced in *psdamt3.* P values are calculated with the Two-sample Kolmogorov-Smirnov test.
(b) In *P. sojae pdamt3* knockout mutant, 6mA levels are reduced around TSS(n=19196). Y-axis is methylation level, calculated as $\log_2(RPKM_{IP}/RPKM_{input})$.
(c)In *P. sojae pdamt3* knockout mutant, 6mA levels are reduced for genes that are located in both GSR (n=3154) and GDR (n=7240). Y-axis is methylation level, calculated as $\log_2(RPKM_{IP}/RPKM_{input})$.

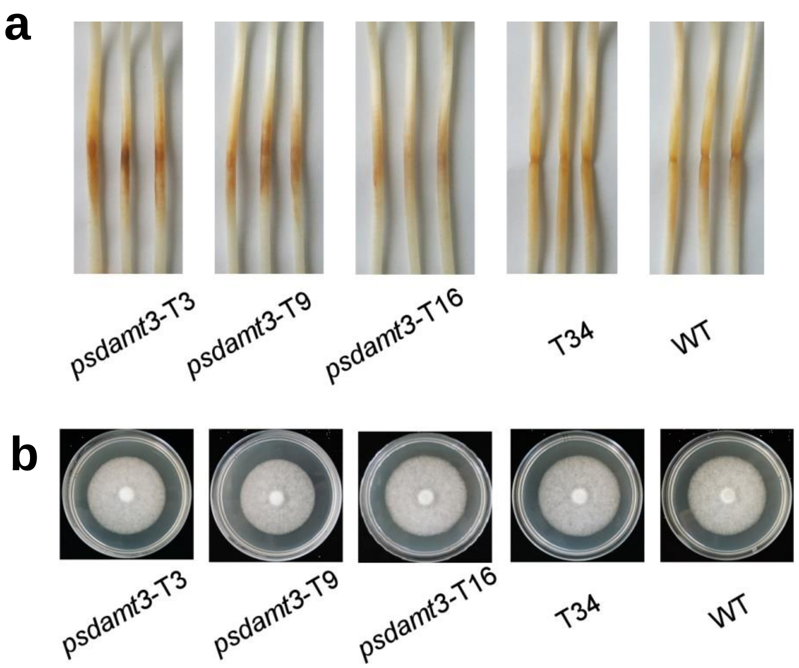

Figure S12
Virulence reduced in *psdamt3* mutants but vegetative growth rate is same.
(a) Etiolated seedlings incubated with zoospores were photographed at 48hpi(hour post inoculation)
(b) Mycelium cultured at V8 medium were photographed at 5dpi(day post inoculation).

a

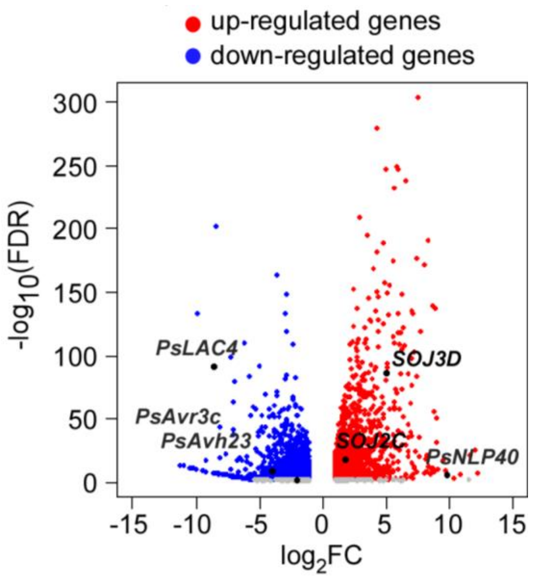

b

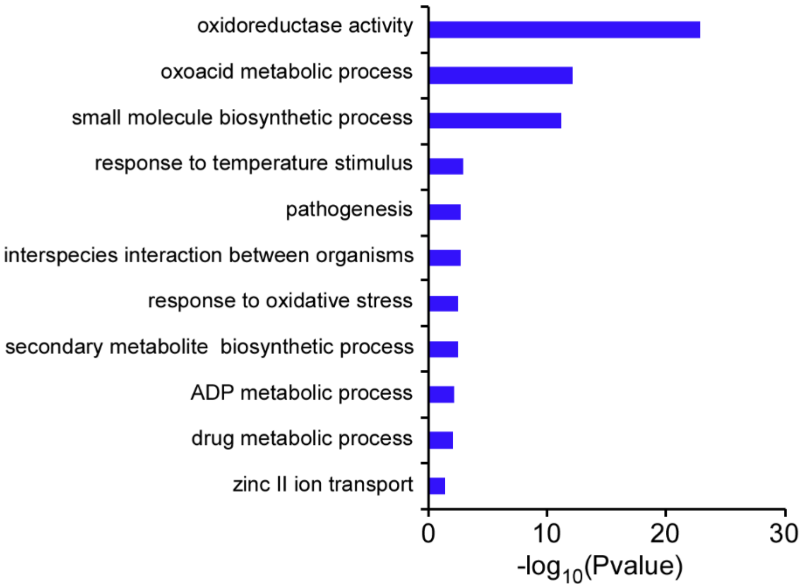

Figure S13

Large scale gene differentially expressed in *psdamt3*

(a) Different expressed genes (DEGs) were showed as volcano plot. X-axis is $\log_2$FC (fold change). y-axis is $-\log_{10}$ (FDR). Fold change calculated as *psdamt3*/WT. Red dots (n=1544) as the up-regulated genes, blue dot (n=1612) as the down-regulated genes. Black dots were representative DEGs between *psdamt3* and WT associated with virulence.

(b) Gene ontology(GO) analysis of DEGs between *psdamt3* and WT. X-axis is $-\log_{10}$(Pvalue), y-axis are selected GO items.

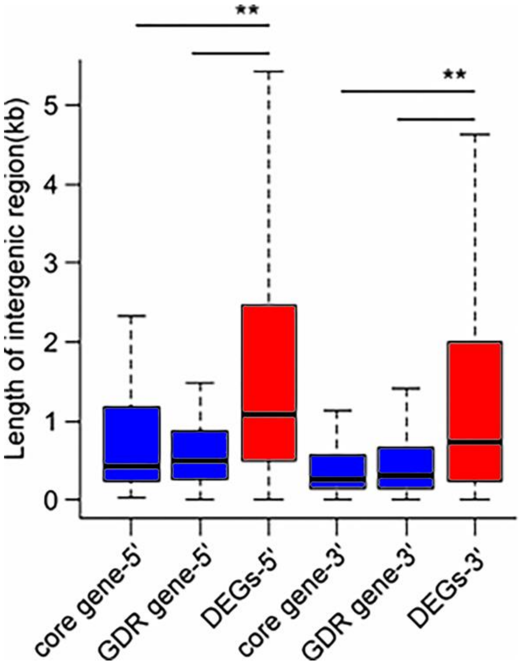

Figure S14

The length of 5’ and 3’ intergenic region of DEGs is longer than core genes and GDR genes. Boxplot showed DEGs (red) have longer 5’ and 3’ intergenic region than core genes (blue) and GDR genes (blue). ** represents significant differences (P<0.01, Two-sample Kolmogorov-Smirnov test)
