# Supplementary material for: Phytophthora methylomes are modulated by 6mA methyltransferases and associated with adaptive genome regions
Source: Genome Biol. 2018 Oct 31;19:181. doi: 10.1186/s13059-018-1564-4 (PMC6211444; doi:10.1186/s13059-018-1564-4)
Supplement: Supplementary file 4 — Supplementary materials. PDF document with supplementary tables. (PDF 24 kb) [file 13059_2018_1564_MOESM4_ESM.pdf]

Table S3 The 6mA quantification data in *P. sojae* and *P. infestans* using UPLC-ESI-MS/MS by three replicates.

Sample	Rep1(p.p.m)	Rep2(p.p.m)	Rep3(p.p.m)	average	STDEV	%*P. sojae* WT
P. sojae	337.58406	454.60349	448.41423	413.53393	65.84	100
P. infestans	550.23693	648.71949	308.99527	502.65056	174.78	121.5504

Table S5 A brief summarize of the mutations of each *psdamt1, psdamt2, psdamt3* mutant.

Mutant	Mode of destruction	Sequence change
psdamt1-T21	Frame shift	-139bp(homozygote)
psdamt1-T50	Frame shift	-1bp(homozygote)
psdamt1-T115	Frame shift	-5bp/-1bp
psdamt2-T10	HDR mediated replacement	RFP replace(homozygote)
psdamt2-T18	HDR mediated replacement	RFP replace(homozygote)
psdamt2-T52	Frame shift	-1bp(homozygote)
psdamt3-T3	Frame shift	-373bp(homozygote)
psdamt3-T9	Frame shift	-374bp(homozygote)
psdamt3-T16	Frame shift	Insertion, +1bp(homozygote)

Table S6 6mA quantification data in *P. sojae DAMT* mutants using UPLC-ESI-MS/MS by three replicates.

Sample	Rep1(p.p.m)	Rep2(p.p.m)	Rep3(p.p.m)	Average	STDEV	%*P. sojae* WT
psdamt1-T21	10.84669	49.71185	40.20488	33.58781	20.25	8.122%
psdamt2-T52	19.38118	26.94702	25.27419	23.86746	3.97	5.771%
psdamt3-T9	62.84280	31.04520	42.37782	45.42194	16.11	10.983%

Table S7 Total normalized read counts at different TE categories

	**DNA elements**	**LTR elements**	**all TE**
WT	38508.67	66927.42	117867.3
psdamt3	56305.21	58578.37	122564.7
